# Supplementary figures and images for: Social bonding in groups of humans selectively increases inter-status information exchange and prefrontal neural synchronization
Source: PLoS Biol. 2024 Mar 19;22(3):e3002545. doi: 10.1371/journal.pbio.3002545 (PMC10950240; doi:10.1371/journal.pbio.3002545)

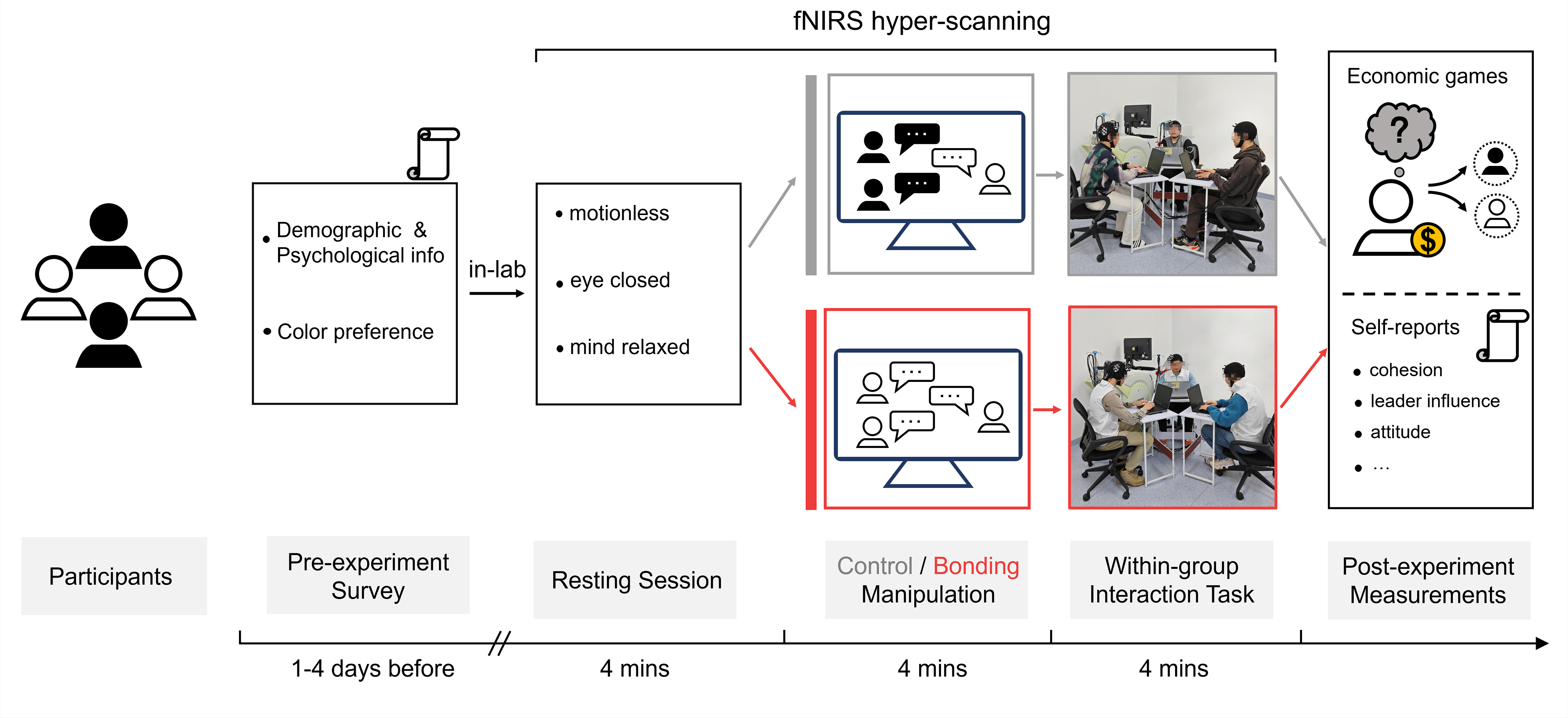

Supplement: S1 Fig — Before coming to the laboratory, participants completed an online survey that included demographic and psychological information as well as color preference (white versus black, for bonding manipulation). One to 4 days later, participants were invited to the laboratory in groups of 3 same-gender strangers and randomly assigned into either the bonding or control condition. They were instructed to sit face-to-face in a triangle and completed 3 sessions during fNIRS-based hyper-scanning: (i) a 4-min resting-state session; (ii) a 4-min in-group social bonding (or no-bonding control) manipulation session; and (iii) a 4-min online within-group interaction session. At the end of the experiment, participants were asked to complete a series of intergroup-related economic games (including intergroup dictator game and intergroup prisoner’s dilemma-maximizing differences game), as well as report subjective evaluations on group cohesion, leader influence and attraction, positive attitudes, willingness to become the leader, etc. (details in Methods). (TIF) [file pbio.3002545.s002.tif]

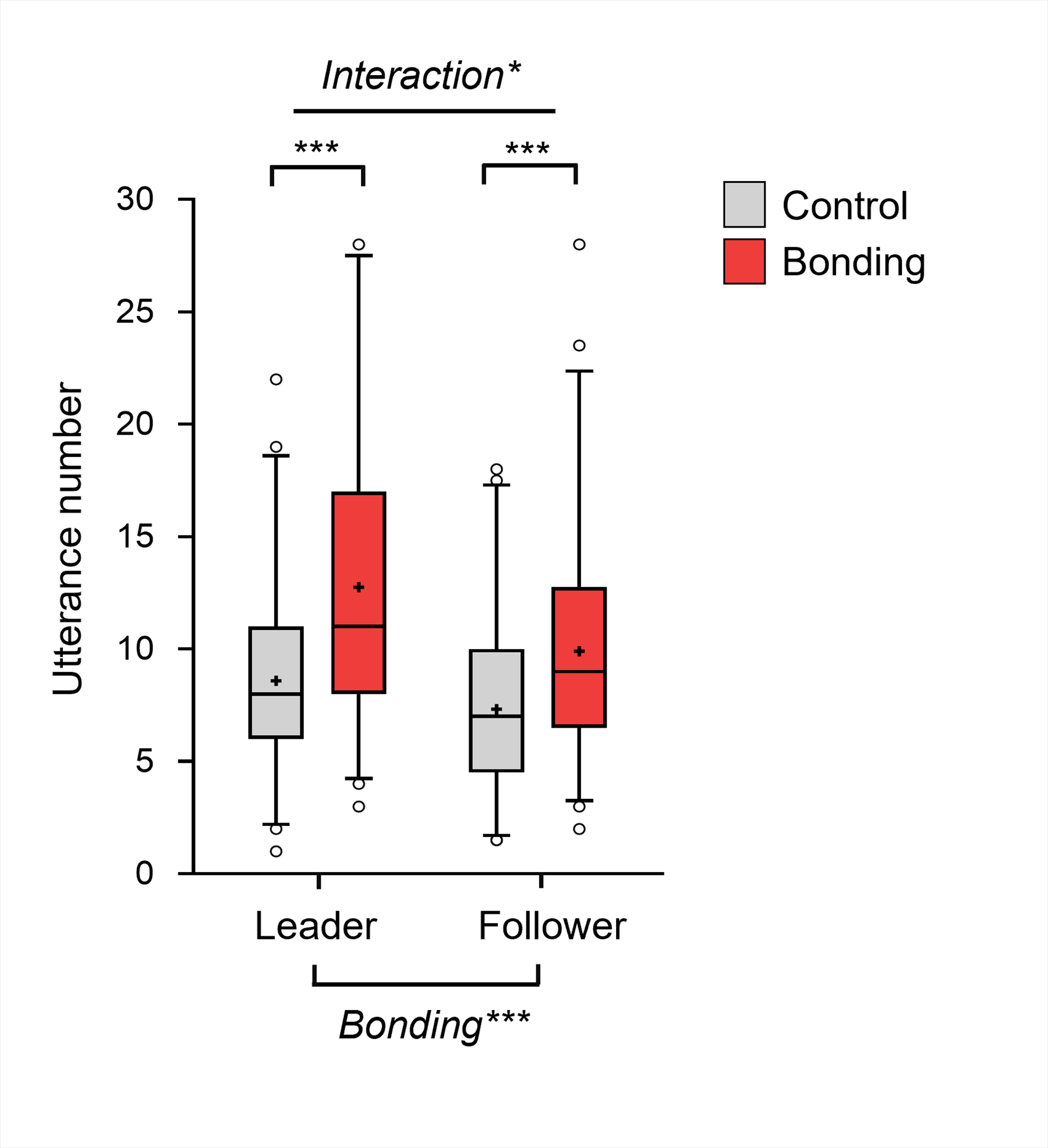

Supplement: S2 Fig — Social bonding particularly increased the utterance numbers given by leaders (control: 8.590 ± 4.088, bonding: 12.750 ± 5.911) than followers (control: 7.322 ± 3.655, bonding: 9.899 ± 4.311). Data are plotted as box plots for each condition, with horizontal lines indicating median values, boxes indicating 25% and 75% quartiles and whiskers indicating the 2.5%–97.5% percentile range. Cross symbols in each box represent the mean values. Data points outside the range are shown separately as circles. *p < 0.05, ***p < 0.001. (TIF) [file pbio.3002545.s003.tif]

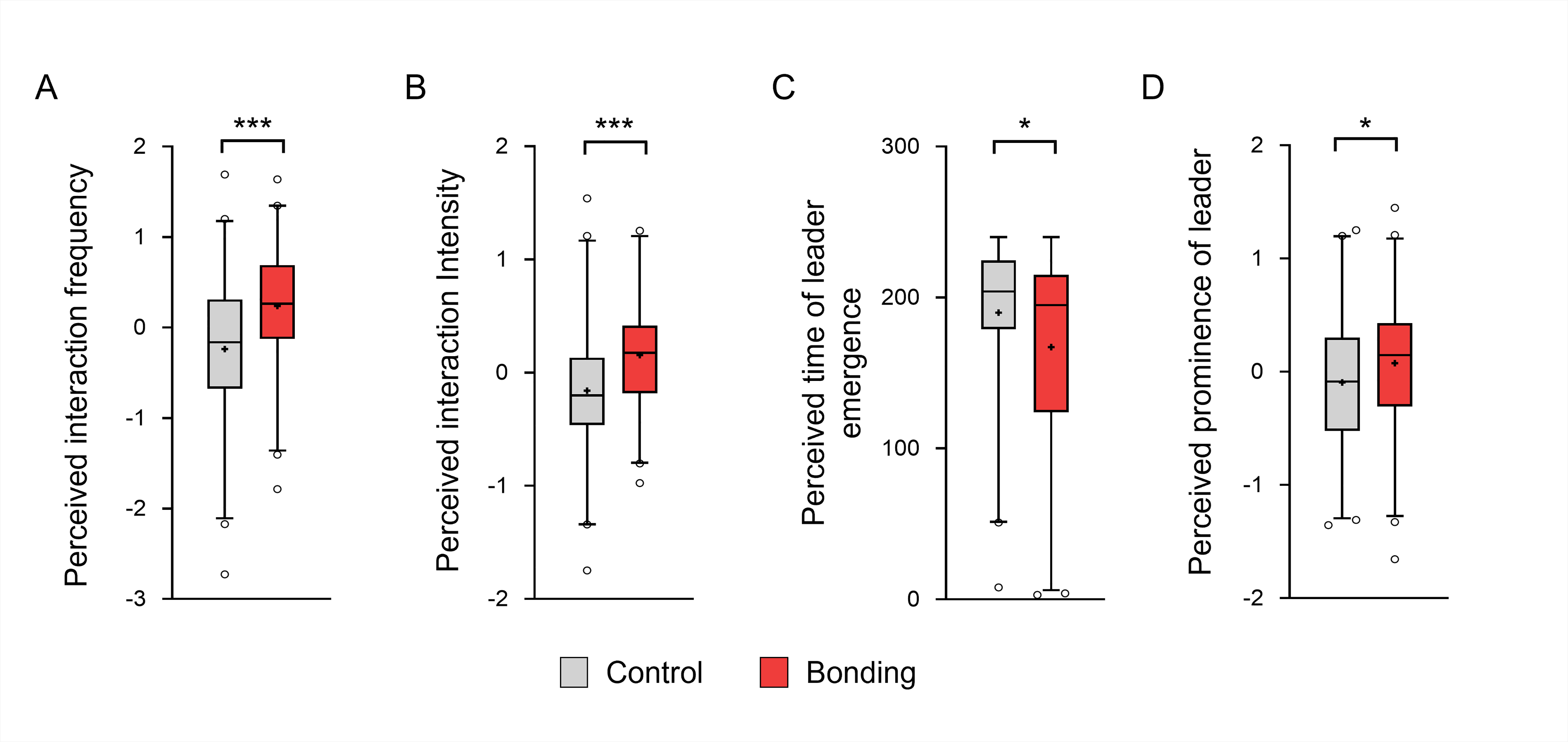

Supplement: S3 Fig — Social bonding increased the perceived group interaction frequency (control: −0.237 ± 0.787, bonding: 0.240 ± 0.629, A) and intensity (control: −0.158 ± 0.542, bonding: 0.156 ± 0.512, B). Third-party observers identified the group leader faster (control: 189.988 ± 49.554, bonding: 167.157 ± 67.349 C), and perceived the leader as more prominent (control: −0.0095 ± 0.582, bonding: 0.076 ± 0.547, D) in the bonding condition. Data are plotted as box plots for each condition, with horizontal lines indicating median values, boxes indicating 25% and 75% quartiles and whiskers indicating the 2.5%–97.5% percentile range. Cross symbols in each box represent the mean values. Data points outside the range are shown separately as circles. *p < 0.05, ***p < 0.001. (TIF) [file pbio.3002545.s004.tif]

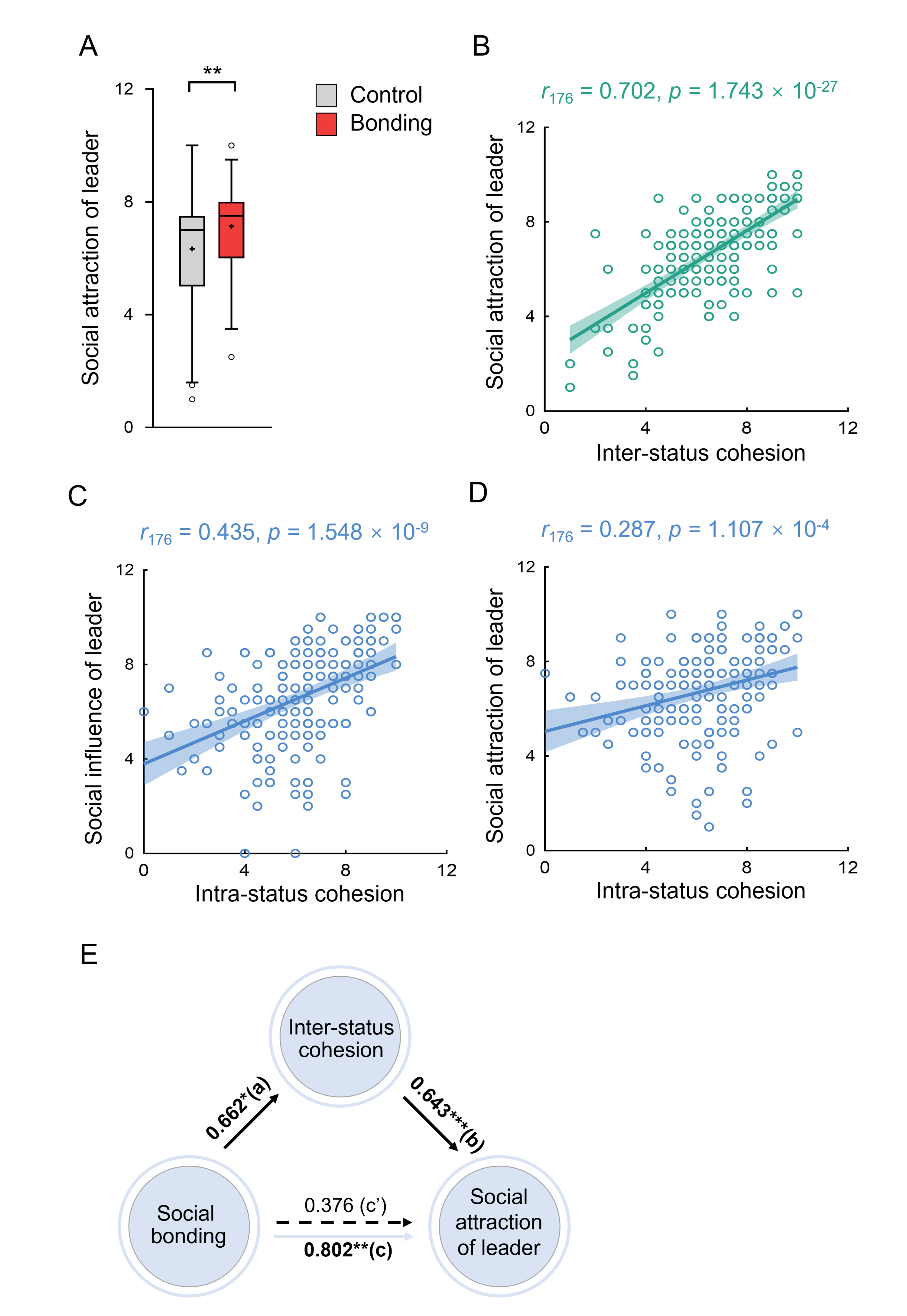

Supplement: S4 Fig — (A) Under social bonding, followers perceived greater social attraction of the leader (control: 6.333 ± 2.036, bonding: 7.135 ± 1.548). (B) Leader’s social attraction was positively associated with inter-status cohesion (Pearson’s correlation analysis). Each solid line represents the least squares fit, with shading showing the 95% CI. (C/D) Leader’s social influence (C) and attraction (D) were positively associated with intra-status cohesion. (E) Bonding increased perceived social attraction of the leader through enhancing inter-status cohesion. *p < 0.05, **p < 0.01, ***p < 0.001. (TIF) [file pbio.3002545.s005.tif]

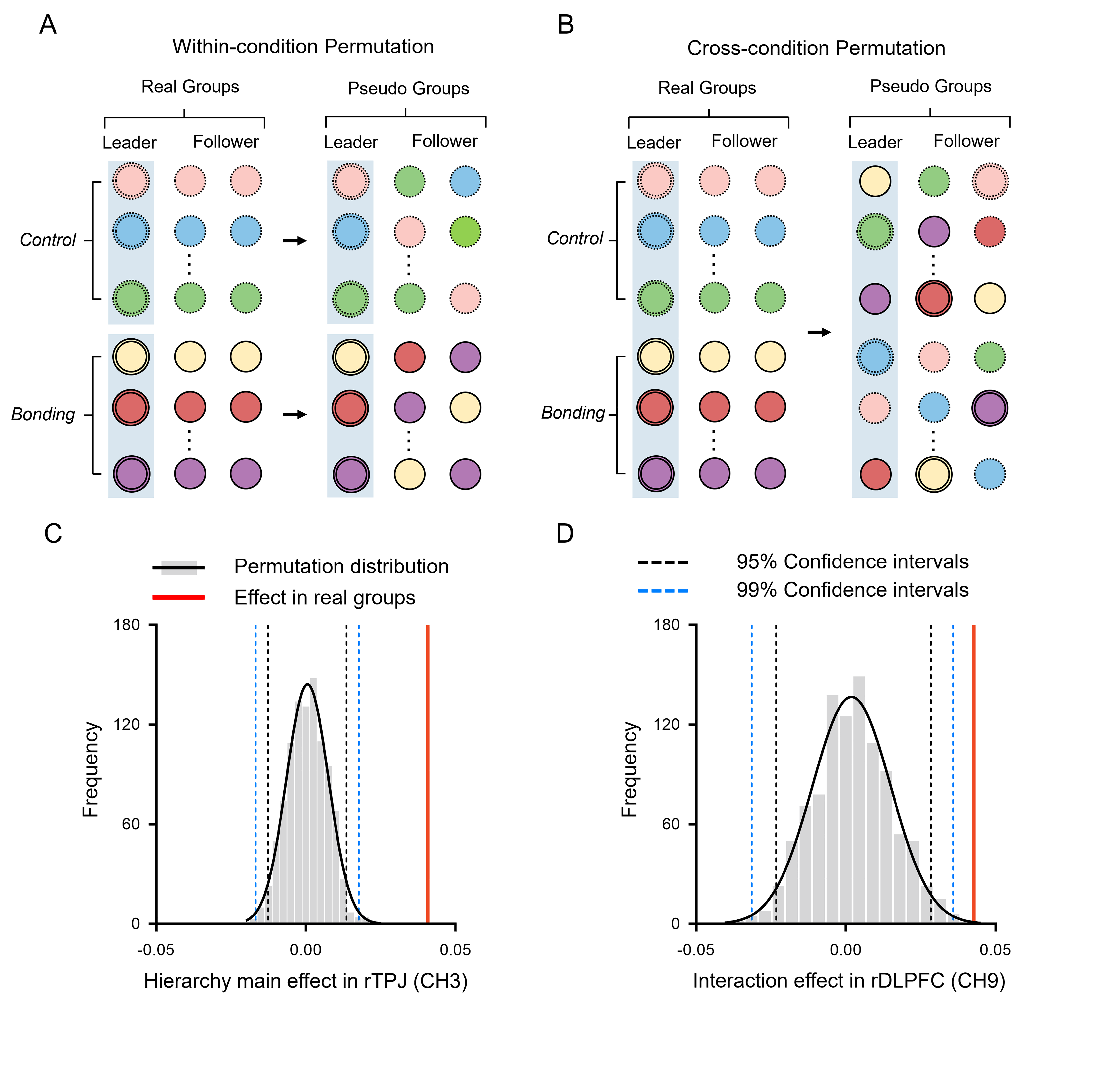

Supplement: S5 Fig — (A/B) We generated within-condition pseudo-groups by randomly grouping a real leader and 2 real followers from different original groups in the same bonding or control condition to 1 pseudo-group (A), or generate across-condition pseudo-groups by randomly grouping 1 leader and 2 followers across bonding and control conditions as one pseudo-group (B). The inter- and intra-status INS for each pseudo group were recalculated. These procedures were repeated for 1,000 times to generate permutation distributions. (C/D) We compared the hierarchy main effect in the rTPJ and the interaction effect in the rDLPFC of real group against cross-condition permutation distributions (n = 1,000). The observed effects of Hierarchy in the rTPJ (C) and of Hierarchy × Bonding interaction in the rDLPFC (D) exceeded the upper limits of 99% CI of the permutation distributions. (TIF) [file pbio.3002545.s006.tif]

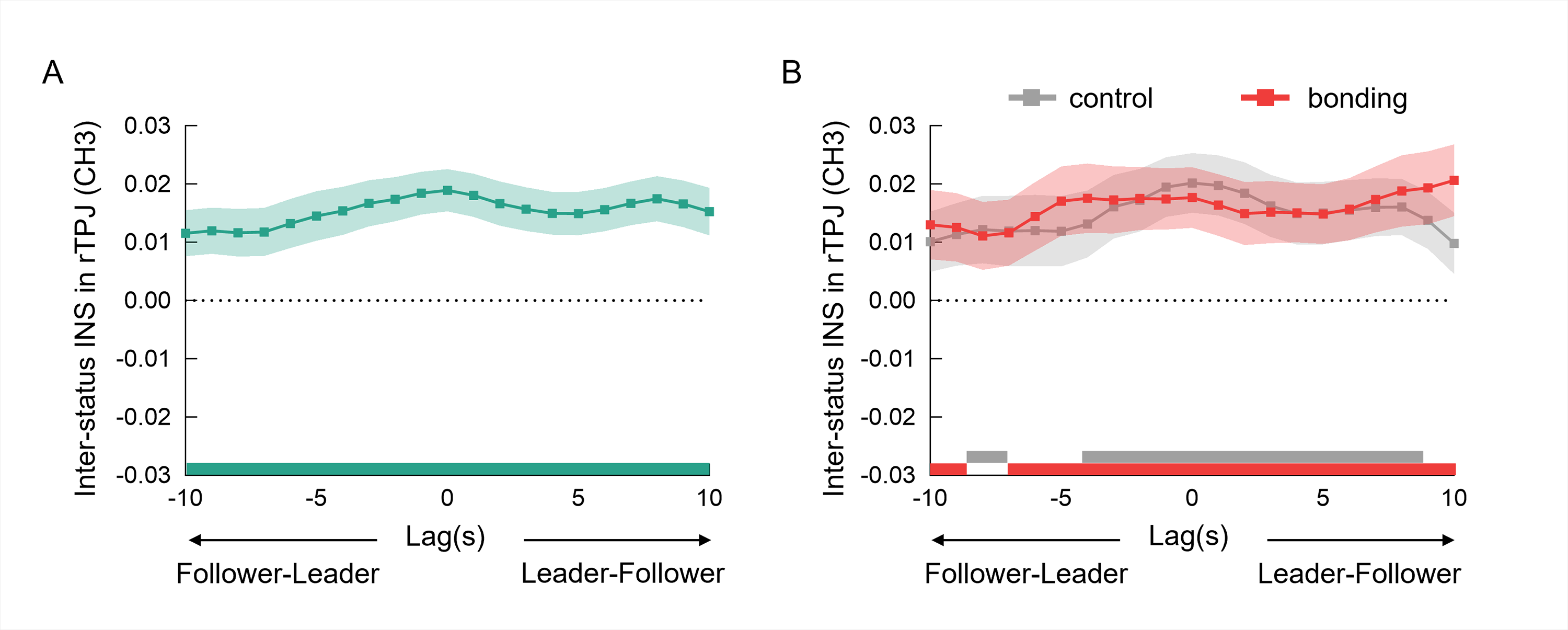

Supplement: S6 Fig — (A) Inter-status neural alignment in rTPJ is significant from −10 to +10 time lags (peaked at 0 s), survived FDR multiple correction. The significant time lags (survived multiple correction) are highlighted with the horizontal line on the x-axis. (B) The inter-status neural alignment in rTPJ is significant at all time lags in bonding and control conditions separately. Shaded areas represent standard error (SE). (TIF) [file pbio.3002545.s007.tif]

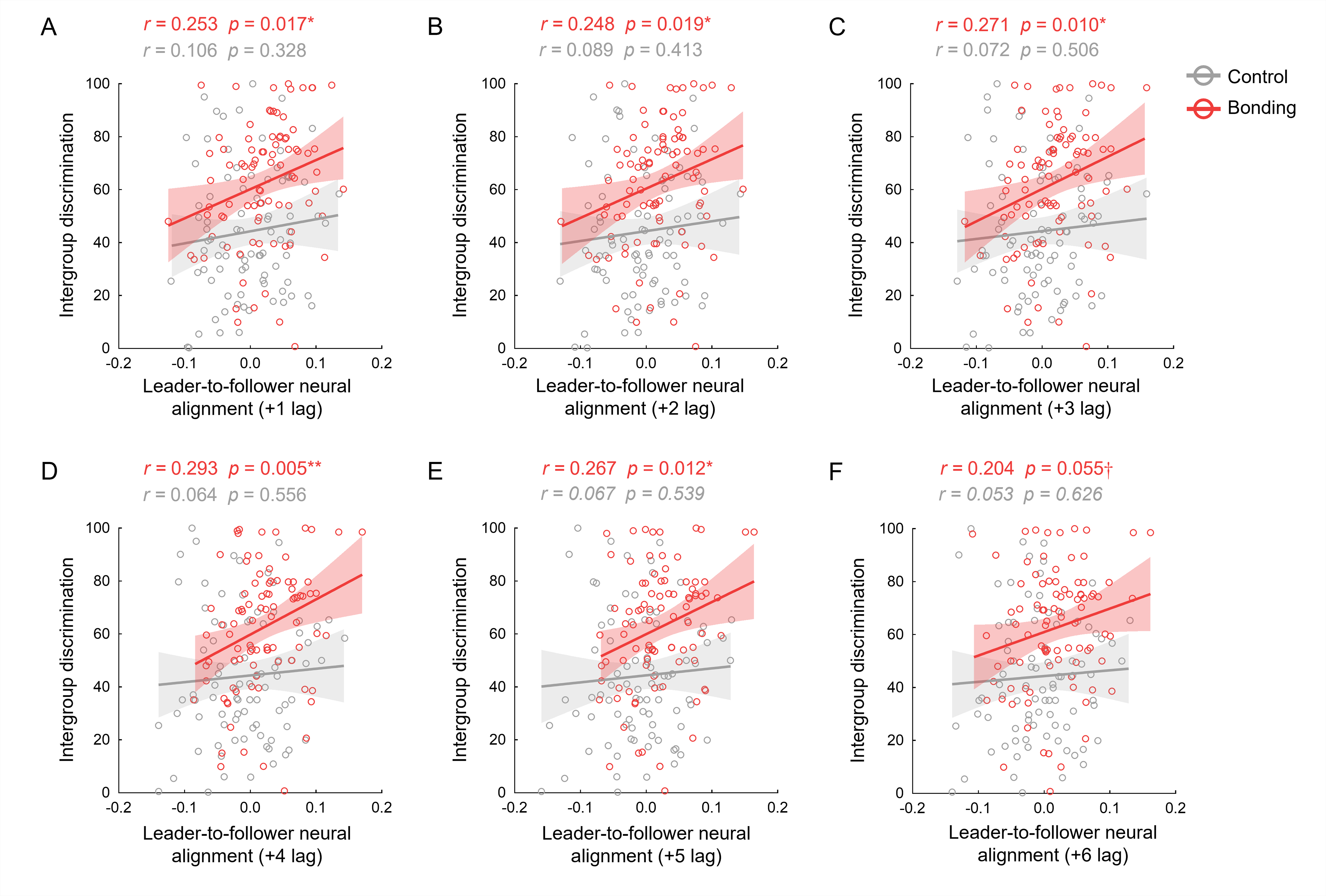

Supplement: S7 Fig — (A–F) Correlation analyses between leader-to-follower neural alignment at each time lag (+1 to +6) with intergroup discrimination. Correlations were performed by Pearson’s correlation coefficient analysis. Each solid line represents the least squares fit, with shading showing the 95% CI. † p < 0.06, *p < 0.05, **p < 0.01. (TIF) [file pbio.3002545.s008.tif]

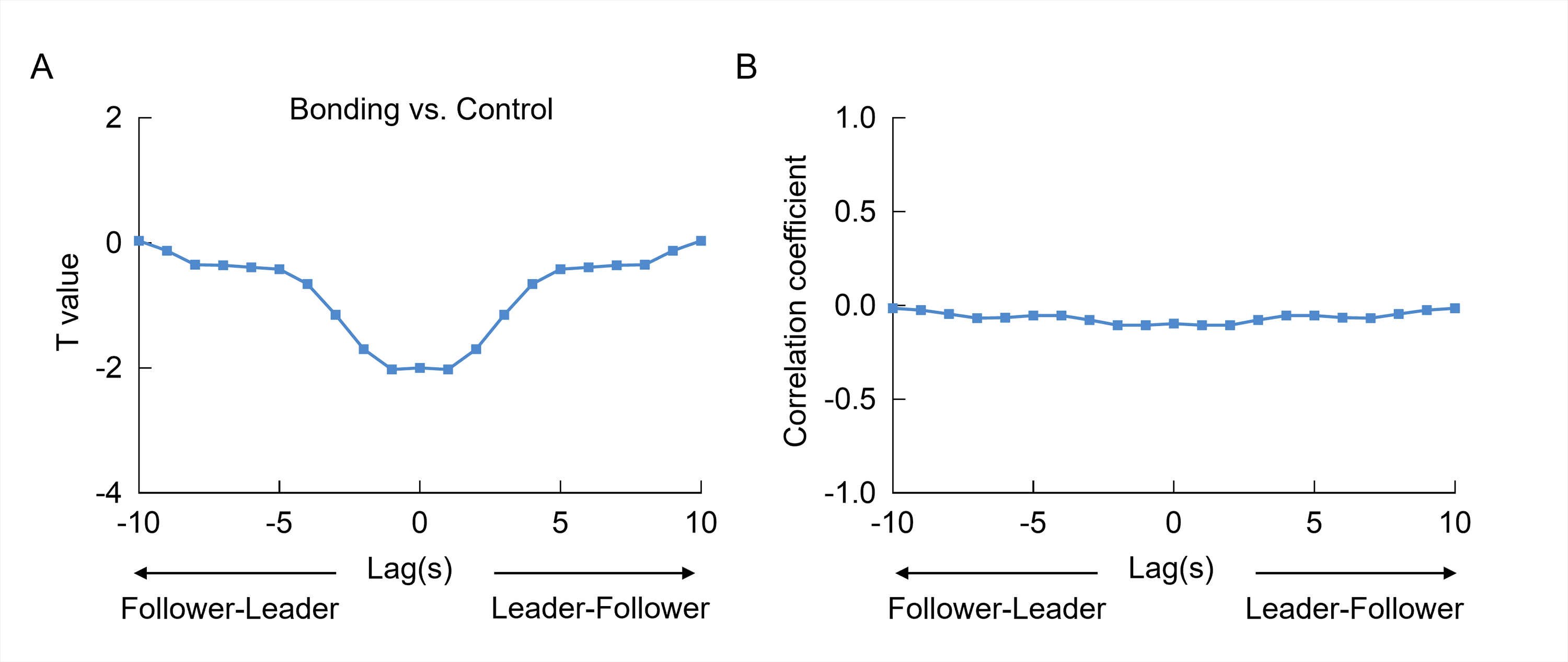

Supplement: S8 Fig — (A) In-group social bonding showed no significant effect on intra-status neural alignment in rDLFPC at any time lags. (B) Intra-status neural alignment in rDLFPC did not correlate with intergroup discrimination at any time lags. (TIF) [file pbio.3002545.s009.tif]

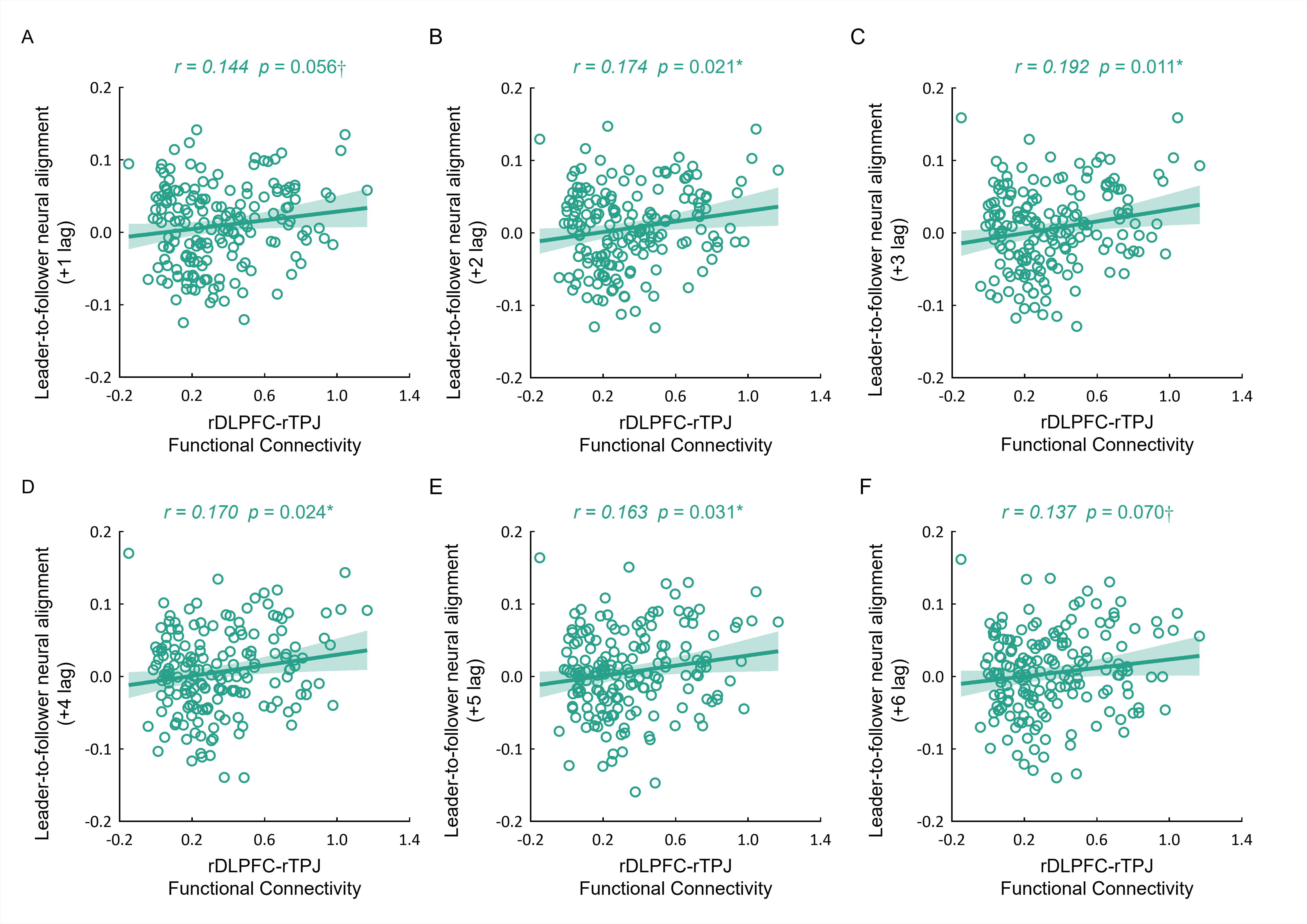

Supplement: S9 Fig — (A–F) Correlation analyses between leaders’ rDLPFC-rTPJ connectivity with leader-to-follower neural alignment at each time lag (+1 to +6). Correlations were performed by Pearson’s correlation coefficient analysis. Each solid line represents the least squares fit, with shading showing the 95% CI. † p < 0.07, *p < 0.05. (TIF) [file pbio.3002545.s010.tif]

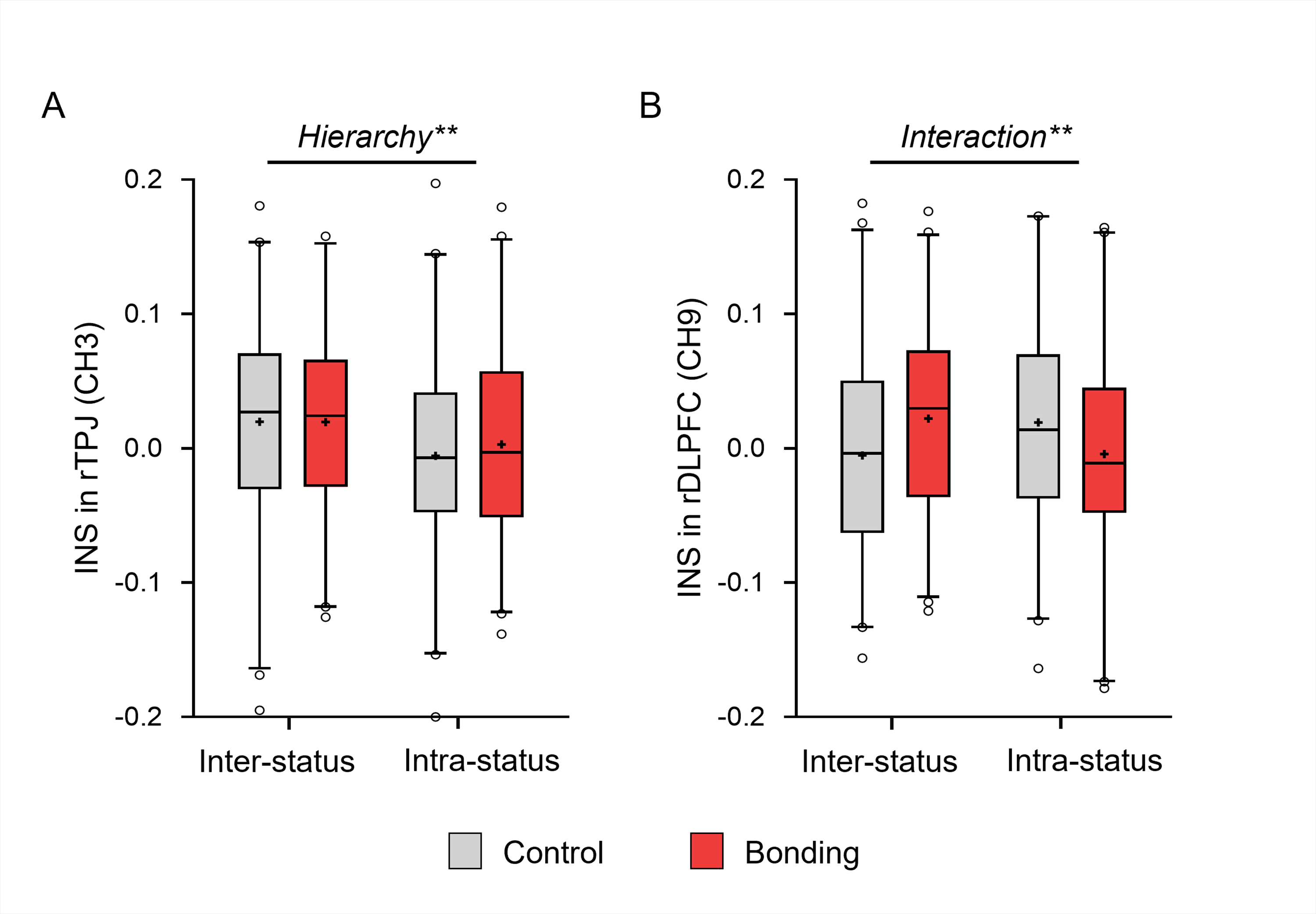

Supplement: S10 Fig — The significant hierarchy main effect in the rTPJ (A) and bonding × hierarchy interaction effect in the rDLPFC (B) were fully replicated when considering inter-status INS for the leader and a randomly selected follower (channel 3, F1, 174 = 8.330, p = 0.004, η2 = 0.046, A; channel 9, F1, 174 = 11.133, p = 0.001, η2 = 0.060, B). Data are plotted as box plots for each condition, with horizontal lines indicating median values, boxes indicating 25% and 75% quartiles and whiskers indicating the 2.5%–97.5% percentile range. Cross symbols in each box represent the mean values. Data points outside the range are shown separately as circles. **p < 0.01. (TIF) [file pbio.3002545.s011.tif]

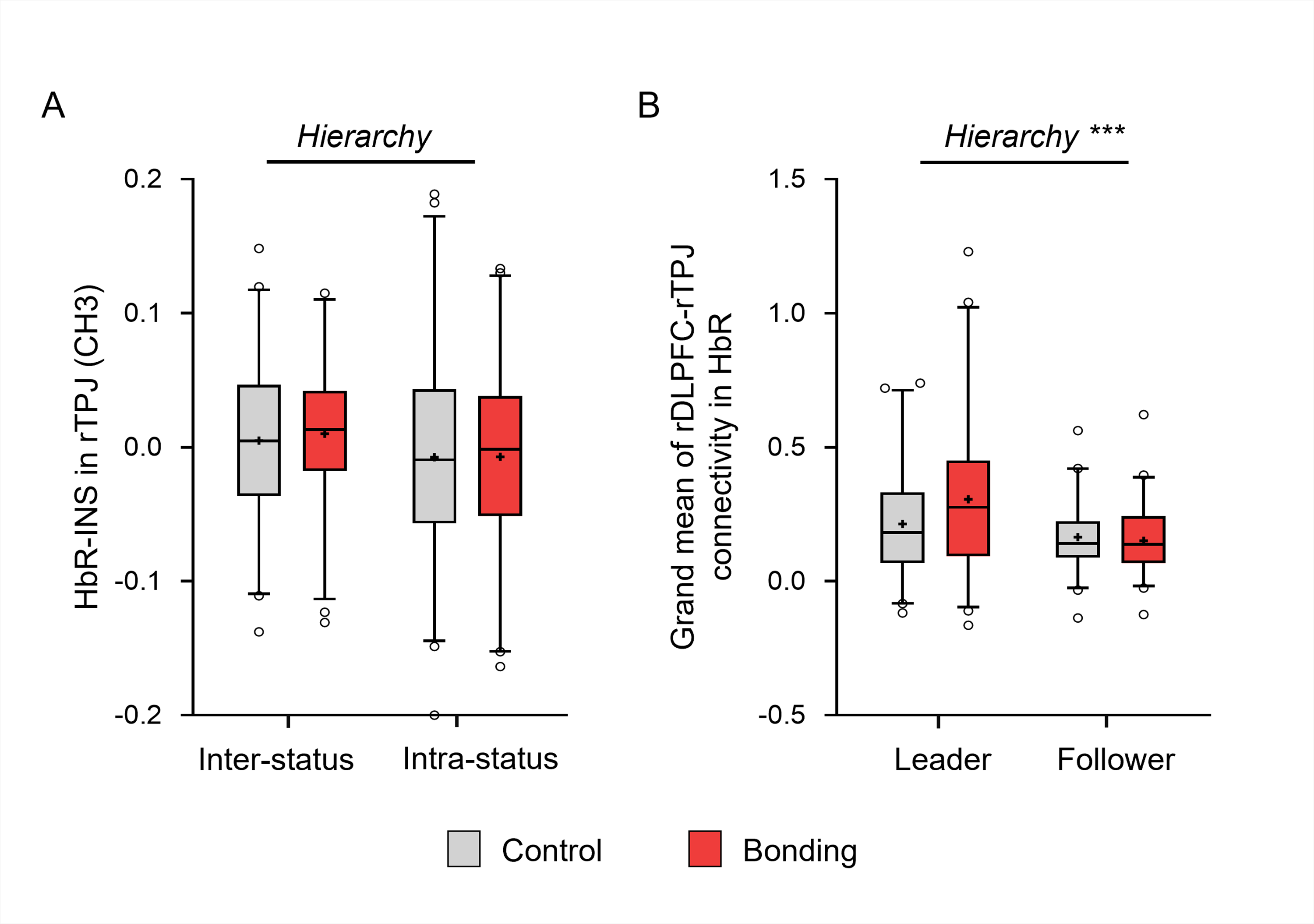

Supplement: S11 Fig — (A) HbR-INS in rTPJ showed a significant, but weaker (did not survive multiple corrections), hierarchy main effect (channel 3, F1, 174 = 4.736, uncorrected p = 0.031, η2 = 0.026), with stronger inter-status INS than intra-status one. (B) The grand mean of rDLPFC-rTPJ connectivity in HbR exhibited a significant hierarchy main effect (F1, 174 = 27.345, p = 4.842 × 10^−7^, η2 = 0.136), with stronger rDLPFC-rTPJ functional connectivity in leaders (vs. followers). Data are plotted as box plots for each condition, with horizontal lines indicating median values, boxes indicating 25% and 75% quartiles and whiskers indicating the 2.5%–97.5% percentile range. Cross symbols in each box represent the mean values. Data points outside the range are shown separately as circles. ***p < 0.001. (TIF) [file pbio.3002545.s012.tif]
