# Supplementary material for: Social bonding in groups of humans selectively increases inter-status information exchange and prefrontal neural synchronization
Source: PLoS Biol. 2024 Mar 19;22(3):e3002545. doi: 10.1371/journal.pbio.3002545 (PMC10950240; doi:10.1371/journal.pbio.3002545)
Supplement: S1 Table — (DOCX) [file pbio.3002545.s013.docx]

**S1 Table. Demographic and psychological information of participants.**

| Measurement | Control | | Bonding | | Bonding | Hierarchy | Interaction |
| --- | --- | --- | --- | --- | --- | --- | --- |
|  | Leader | Follower | Leader | Follower |  |  |  |
|  | Mean (SE) | Mean (SE) | Mean (SE) | Mean (SE) | *p* | *p* | *p* |
| - **Demographic information** | | | | | | | |
| Number (*N*) | 87 | 87 | 89 | 89 | - | - | - |
| Gender (*N_male_* vs. *N_female_*) | 37 vs. 50 | 37 vs. 50 | 43 vs. 46 | 43 vs. 46 | - | - | - |
| Age (year) | 21.40 (0.27) | 21.95 (0.19) | 22.14 (0.29) | 22.16 (0.21) | 0.07 | 0.21 | 0.24 |
| Education year | 16.52 (0.18) | 16.69 (0.14) | 16.92 (0.22) | 16.96 (0.15) | 0.06 | 0.55 | 0.70 |
| - **Psychological information** | | | | | | | |
| Empathic capacity | 2.38 (0.03) | 2.36 (0.03) | 2.38 (0.03) | 2.35 (0.03) | 0.88 | 0.85 | 0.48 |
| Cooperative personality | 3.81 (0.05) | 3.80 (0.04) | 3.84 (0.05) | 3.87 (0.04) | 0.22 | 0.85 | 0.67 |
| Social value orientation | 26.28 (1.38) | 25.52 (0.97) | 25.59 (1.53) | 26.48 (1.05) | 0.82 | 0.94 | 0.59 |
| Prosocial personality | 3.15 (0.04) | 3.19 (0.03) | 3.15 (0.04) | 3.20 (0.04) | 0.23 | 0.96 | 0.89 |
| Justice sensitivity | 2.76 (0.07) | 2.79 (0.06) | 2.69 (0.07) | 2.73 (0.05) | 0.32 | 0.57 | 0.99 |
| Preference for social hierarchy hiehierarchy | 3.43 (0.08) | 3.31 (0.06) | 3.40 (0.09) | 3.41 (0.06) | 0.62 | 0.48 | 0.46 |
| Intergroup discrimination (IDG) | 34.52 (3.83) | 34.23 (2.86) | 35.81 (4.23) | 37.02 (2.82) | 0.56 | 0.90 | 0.83 |
| Ingroup love (IPD-MD) | 43.36 (3.21) | 41.44 (1.91) | 41.78 (3.44) | 46.28 (2.40) | 0.55 | 0.66 | 0.27 |
| Outgroup hate (IPD-MD) | 20.88 (2.45) | 23.91 (1.62) | 24.43 (3.11) | 24.74 (2.13) | 0.36 | 0.49 | 0.57 |
| Independence (SCS) | 5.03(0.07) | 5.05(0.05) | 5.16(0.07) | 4.98(0.06) | 0.58 | 0.24 | 0.15 |
| Interdependence (SCS) | 5.06(0.07) | 5.15(0.05) | 5.14(0.07) | 5.08(0.05) | 0.96 | 0.81 | 0.24 |
