# Supplementary material for: Social bonding in groups of humans selectively increases inter-status information exchange and prefrontal neural synchronization
Source: PLoS Biol. 2024 Mar 19;22(3):e3002545. doi: 10.1371/journal.pbio.3002545 (PMC10950240; doi:10.1371/journal.pbio.3002545)
Supplement: S2 Table — (DOCX) [file pbio.3002545.s014.docx]

**S2 Table. Full statistical reports of the results of Hierarchy × Bonding mixed-model ANOVAs on inter-brain neural synchronization.**

| Channel | Effect | *F* | *p* | *η^2^* | FDR-corrected *p* |
| --- | --- | --- | --- | --- | --- |
| ***TPJ*** |  |  |  |  |  |
| 1 | Bonding | 1.102 | 0.295 | 0.006 | 0.998 |
|  | Hierarchy | 0.075 | 0.785 | 4.29×10^-4^ | 0.785 |
|  | Bonding × Hierarchy | 0.139 | 0.710 | 0.001 | 0.919 |
|  |  |  |  |  |  |
| 2 | Bonding | 0.002 | 0.961 | 1.39×10^-5^ | 0.998 |
|  | Hierarchy | 2.400 | 0.123 | 0.014 | 0.575 |
|  | Bonding × Hierarchy | 0.073 | 0.788 | 4.17×10^-4^ | 0.919 |
|  |  |  |  |  |  |
| 3 | Bonding | 0.200 | 0.656 | 0.001 | 0.998 |
|  | **Hierarchy**** | **10.207** | **0.002** | **0.055** | **0.023** |
|  | Bonding × Hierarchy | 0.747 | 0.389 | 0.004 | 0.719 |
|  |  |  |  |  |  |
| 4 | Bonding | 0.420 | 0.518 | 0.002 | 0.998 |
|  | Hierarchy | 0.128 | 0.721 | 0.001 | 0.785 |
|  | Bonding × Hierarchy | 0.680 | 0.411 | 0.004 | 0.719 |
|  |  |  |  |  |  |
| 5 | Bonding | 0.172 | 0.679 | 0.001 | 0.998 |
|  | Hierarchy | 1.246 | 0.266 | 0.007 | 0.679 |
|  | Bonding × Hierarchy | 3.922 | 0.049 | 0.022 | 0.345 |
|  |  |  |  |  |  |
| 6 | Bonding | 1.548 | 0.215 | 0.009 | 0.998 |
|  | Hierarchy | 1.123 | 0.291 | 0.006 | 0.679 |
|  | Bonding × Hierarchy | 3.108 | 0.080 | 0.018 | 0.372 |
|  |  |  |  |  |  |
| 7 | Bonding | 4.513 | 0.035 | 0.025 | 0.491 |
|  | Hierarchy | 0.114 | 0.736 | 0.001 | 0.785 |
|  | Bonding × Hierarchy | 1.746 | 0.188 | 0.010 | 0.658 |
| ***DLPFC*** |  |  |  |  |  |
| 8 | Bonding | 0.020 | 0.887 | 1.17×10^-4^ | 0.998 |
|  | Hierarchy | 0.164 | 0.686 | 0.001 | 0.785 |
|  | Bonding × Hierarchy | 0.023 | 0.879 | 1.34×10^-4^ | 0.946 |
|  |  |  |  |  |  |
| 9 | Bonding | 0.084 | 0.773 | 4.81×10^-4^ | 0.998 |
|  | Hierarchy | 0.128 | 0.721 | 0.001 | 0.785 |
|  | **Bonding × Hierarchy**** | **9.577** | **0.002** | **0.052** | **0.032** |
|  |  |  |  |  |  |
| 10 | Bonding | 7.88×10^-6^ | 0.998 | 4.53×10^-8^ | 0.998 |
|  | Hierarchy | 0.873 | 0.351 | 0.005 | 0.703 |
|  | Bonding × Hierarchy | 0.093 | 0.761 | 0.001 | 0.919 |
|  |  |  |  |  |  |
| 11 | Bonding | 0.072 | 0.789 | 4.14×10^-4^ | 0.998 |
|  | Hierarchy | 2.631 | 0.107 | 0.015 | 0.575 |
|  | Bonding × Hierarchy | 0.002 | 0.962 | 1.31×10^-5^ | 0.962 |
|  |  |  |  |  |  |
| 12 | Bonding | 0.535 | 0.465 | 0.003 | 0.998 |
|  | Hierarchy | 0.342 | 0.560 | 0.002 | 0.785 |
|  | Bonding × Hierarchy | 1.378 | 0.242 | 0.008 | 0.678 |
|  |  |  |  |  |  |
| 13 | Bonding | 0.447 | 0.505 | 0.003 | 0.998 |
|  | Hierarchy | 1.573 | 0.211 | 0.009 | 0.679 |
|  | Bonding × Hierarchy | 0.702 | 0.403 | 0.004 | 0.719 |
|  |  |  |  |  |  |
| 14 | Bonding | 0.688 | 0.408 | 0.004 | 0.998 |
|  | Hierarchy | 0.139 | 0.710 | 0.001 | 0.785 |
|  | Bonding × Hierarchy | 0.102 | 0.750 | 0.001 | 0.919 |

Note: ****** *p* < 0.01, FDR corrected.
