# Supplementary material for: Social bonding in groups of humans selectively increases inter-status information exchange and prefrontal neural synchronization
Source: PLoS Biol. 2024 Mar 19;22(3):e3002545. doi: 10.1371/journal.pbio.3002545 (PMC10950240; doi:10.1371/journal.pbio.3002545)
Supplement: S3 Table — (A) ANOVA analysis after the wavelet-based denoising. (B) ANCOVA analysis controlling global mean INS. (DOCX) [file pbio.3002545.s015.docx]

**S3 Table. Statistical reports of inter-brain neural synchronization in two complementary analyses.**

1. **ANOVA analysis after the wavelet-based denoising**

| Channel | Effect | *F* | *p* | *η^2^* |
| --- | --- | --- | --- | --- |
| ***TPJ*** |  |  |  |  |
| 3 | Bonding | 0.002 | 0.961 | 1.40×10^-5^ |
|  | **Hierarchy*** | **6.296** | **0.013** | **0.035** |
|  | Bonding ×Hierarchy | 0.607 | 0.437 | 0.003 |
| ***DLPFC*** |  |  |  |  |
| 9 | Bonding | 0.254 | 0.615 | 0.001 |
|  | Hierarchy | 0.119 | 0.731 | 0.001 |
|  | **Bonding × Hierarchy*** | **5.311** | **0.022** | **0.030** |

1. **ANCOVA analysis controlling global mean INS**

| Channel | Effect | *F* | *p* | *η^2^* |
| --- | --- | --- | --- | --- |
| ***TPJ*** |  |  |  |  |
| 3 | Bonding | 0.232 | 0.631 | 0.001 |
|  | **Hierarchy**** | **9.220** | **0.003** | **0.051** |
|  | Bonding ×Hierarchy | 1.308 | 0.254 | 0.008 |
| ***DLPFC*** |  |  |  |  |
| 9 | Bonding | 0.053 | 0.818 | 3.10×10^-4^ |
|  | Hierarchy | 0.536 | 0.465 | 0.003 |
|  | **Bonding ×Hierarchy**** | **8.373** | **0.004** | **0.046** |

Note: ***** *p* < 0.05, ****** *p* < 0.01.
