# Supplementary material for: Social bonding in groups of humans selectively increases inter-status information exchange and prefrontal neural synchronization
Source: PLoS Biol. 2024 Mar 19;22(3):e3002545. doi: 10.1371/journal.pbio.3002545 (PMC10950240; doi:10.1371/journal.pbio.3002545)
Supplement: S4 Table — (DOCX) [file pbio.3002545.s016.docx]

**S4 Table. Full statistical reports of the results of bonding effect (Bonding vs. Control) on inter-status neural alignment (CH9) for each time lag.**

| Time Lags | *t* | *p* | *Cohen’s d* | FDR-corrected *p* |
| --- | --- | --- | --- | --- |
| ***Follower-to-Leader*** |  |  |  |  |
| -10 | 0.324 | 0.746 | 0.049 | 0.816 |
| -9 | 0.570 | 0.570 | 0.086 | 0.719 |
| -8 | 0.589 | 0.557 | 0.089 | 0.719 |
| -7 | 0.551 | 0.582 | 0.083 | 0.719 |
| -6 | 0.633 | 0.527 | 0.095 | 0.719 |
| -5 | 0.614 | 0.540 | 0.093 | 0.719 |
| -4 | 0.486 | 0.627 | 0.073 | 0.732 |
| -3 | 0.630 | 0.530 | 0.095 | 0.719 |
| -2 | 0.799 | 0.426 | 0.120 | 0.719 |
| -1 | 1.686 | 0.094 | 0.254 | 0.219 |
| 0 | 2.357 | 0.020 | 0.355 | 0.060 |
| ***Leader-to-Follower*** |  |  |  |  |
| **1**** | **2.858** | **0.005** | **0.431** | **0.018** |
| **2**** | **2.885** | **0.004** | **0.435** | **0.017** |
| **3**** | **3.010** | **0.003** | **0.454** | **0.016** |
| **4**** | **3.314** | **0.001** | **0.500** | **0.011** |
| **5***** | **3.475** | **0.001** | **0.524** | **0.011** |
| **6**** | **2.970** | **0.003** | **0.448** | **0.016** |
| 7 | 1.965 | 0.051 | 0.296 | 0.134 |
| 8 | 0.888 | 0.376 | 0.134 | 0.719 |
| 9 | 0.234 | 0.816 | 0.035 | 0.816 |
| 10 | 0.239 | 0.811 | 0.036 | 0.816 |

Note: ****** *p* < 0.01, ******* *p* < 0.001, FDR corrected.
