# Supplementary material for: Social bonding in groups of humans selectively increases inter-status information exchange and prefrontal neural synchronization
Source: PLoS Biol. 2024 Mar 19;22(3):e3002545. doi: 10.1371/journal.pbio.3002545 (PMC10950240; doi:10.1371/journal.pbio.3002545)
Supplement: S5 Table — (DOCX) [file pbio.3002545.s017.docx]

**S5 Table. Full statistical reports of the results of inter-status INS increase (CH9, one-sample t-tests) of each time lag under bonding and control conditions respectively.**

| Time Lags | Condition | *t* | *p* | *Cohen’s d* | FDR-corrected *p* |
| --- | --- | --- | --- | --- | --- |
| ***Follower-to-Leader*** | |  |  |  |  |
| -10 | **Bonding**** | **2.797** | **0.006** | **0.297** | **0.015** |
|  | Control | 2.570 | 0.012 | 0.276 | 0.252 |
|  |  |  |  |  |  |
| -9 | **Bonding*** | **2.610** | **0.011** | **0.277** | **0.022** |
|  | Control | 1.984 | 0.050 | 0.213 | 0.525 |
|  |  |  |  |  |  |
| -8 | Bonding | 1.928 | 0.057 | 0.204 | 0.078 |
|  | Control | 1.320 | 0.190 | 0.142 | 0.525 |
|  |  |  |  |  |  |
| -7 | Bonding | 1.423 | 0.158 | 0.151 | 0.185 |
|  | Control | 0.810 | 0.420 | 0.087 | 0.630 |
|  |  |  |  |  |  |
| -6 | Bonding | 1.484 | 0.141 | 0.157 | 0.175 |
|  | Control | 0.679 | 0.499 | 0.073 | 0.634 |
|  |  |  |  |  |  |
| -5 | Bonding | 1.911 | 0.059 | 0.203 | 0.078 |
|  | Control | 1.132 | 0.261 | 0.121 | 0.535 |
|  |  |  |  |  |  |
| -4 | Bonding | 1.974 | 0.052 | 0.209 | 0.077 |
|  | Control | 1.458 | 0.149 | 0.156 | 0.525 |
|  |  |  |  |  |  |
| -3 | Bonding | 2.145 | 0.035 | 0.227 | 0.061 |
|  | Control | 1.427 | 0.157 | 0.153 | 0.525 |
|  |  |  |  |  |  |
| -2 | **Bonding*** | **2.267** | **0.026** | **0.240** | **0.049** |
|  | Control | 1.359 | 0.178 | 0.146 | 0.525 |
|  |  |  |  |  |  |
| -1 | **Bonding**** | **2.933** | **0.004** | **0.311** | **0.011** |
|  | Control | 0.656 | 0.513 | 0.070 | 0.634 |
|  |  |  |  |  |  |
| 0 | **Bonding**** | **3.378** | **0.001** | **0.358** | **0.004** |
|  | Control | 0.051 | 0.959 | 0.006 | 0.959 |
| ***Leader-to-Follower*** | |  |  |  |  |
| 1 | **Bonding***** | **3.666** | **4.21×10^-4^** | **0.389** | **0.003** |
|  | Control | -0.472 | 0.638 | -0.051 | 0.705 |
|  |  |  |  |  |  |
| 2 | **Bonding**** | **3.344** | **0.001** | **0.354** | **0.004** |
|  | Control | -0.870 | 0.387 | -0.093 | 0.630 |
|  |  |  |  |  |  |
| 3 | **Bonding **** | **3.334** | **0.001** | **0.353** | **0.004** |
|  | Control | -1.087 | 0.280 | -0.116 | 0.535 |
|  |  |  |  |  |  |
| 4 | **Bonding***** | **3.722** | **3.48×10^-4^** | **0.395** | **0.003** |
|  | Control | -1.202 | 0.233 | -0.129 | 0.535 |
|  |  |  |  |  |  |
| 5 | **Bonding***** | **3.842** | **2.30×10^-4^** | **0.407** | **0.003** |
|  | Control | -1.321 | 0.190 | -0.142 | 0.525 |
|  |  |  |  |  |  |
| 6 | **Bonding**** | **2.997** | **0.004** | **0.318** | **0.011** |
|  | Control | -1.292 | 0.200 | -0.139 | 0.525 |
|  |  |  |  |  |  |
| 7 | Bonding | 2.038 | 0.045 | 0.216 | 0.072 |
|  | Control | -0.756 | 0.452 | -0.081 | 0.633 |
|  |  |  |  |  |  |
| 8 | Bonding | 1.173 | 0.244 | 0.124 | 0.256 |
|  | Control | -0.118 | 0.906 | -0.013 | 0.951 |
|  |  |  |  |  |  |
| 9 | Bonding | 0.976 | 0.332 | 0.103 | 0.332 |
|  | Control | 0.559 | 0.578 | 0.060 | 0.674 |
|  |  |  |  |  |  |
| 10 | Bonding | 1.286 | 0.202 | 0.136 | 0.223 |
|  | Control | 0.816 | 0.417 | 0.087 | 0.630 |

Note: ***** *p* < 0.05, ****** *p* < 0.01, ******* *p* < 0.001, FDR corrected.
