# Supplementary material for: Social bonding in groups of humans selectively increases inter-status information exchange and prefrontal neural synchronization
Source: PLoS Biol. 2024 Mar 19;22(3):e3002545. doi: 10.1371/journal.pbio.3002545 (PMC10950240; doi:10.1371/journal.pbio.3002545)
Supplement: S6 Table — (DOCX) [file pbio.3002545.s018.docx]

**S6 Table. Full statistical reports of the results of the Pearson’s correlations between inter-status neural alignment (CH9) and intergroup discrimination on each time lag.**

| Time Lags | *r* | *p* | FDR-corrected *p* |
| --- | --- | --- | --- |
| ***Follower-to-Leader*** |  |  |  |
| -10 | -0.043 | 0.574 | 0.668 |
| -9 | -0.031 | 0.679 | 0.713 |
| -8 | -0.002 | 0.980 | 0.980 |
| -7 | 0.039 | 0.604 | 0.668 |
| -6 | 0.076 | 0.315 | 0.389 |
| -5 | 0.089 | 0.242 | 0.318 |
| -4 | 0.101 | 0.181 | 0.269 |
| -3 | 0.108 | 0.152 | 0.266 |
| -2 | 0.127 | 0.093 | 0.195 |
| -1 | 0.163 | 0.030 | 0.079 |
| **0*** | **0.216** | **0.004** | **0.014** |
| ***Leader-to-Follower*** |  |  |  |
| **1**** | **0.238** | **0.001** | **0.008** |
| **2**** | **0.227** | **0.002** | **0.008** |
| **3**** | **0.230** | **0.002** | **0.008** |
| **4**** | **0.240** | **0.001** | **0.008** |
| **5**** | **0.234** | **0.002** | **0.008** |
| **6*** | **0.193** | **0.010** | **0.030** |
| 7 | 0.158 | 0.036 | 0.084 |
| 8 | 0.116 | 0.125 | 0.239 |
| 9 | 0.103 | 0.174 | 0.269 |
| 10 | 0.099 | 0.192 | 0.269 |

Note: ***** *p* < 0.05, ****** *p* < 0.01, FDR corrected.
