# Supplementary material for: Social bonding in groups of humans selectively increases inter-status information exchange and prefrontal neural synchronization
Source: PLoS Biol. 2024 Mar 19;22(3):e3002545. doi: 10.1371/journal.pbio.3002545 (PMC10950240; doi:10.1371/journal.pbio.3002545)
Supplement: S7 Table — (DOCX) [file pbio.3002545.s019.docx]

**S7 Table. Full statistical reports of hierarchy main effect on rDLPFC-rTPJ functional connectivity for each channel pair.**

| Channel pairs  (rDLPFC-rTPJ) | *F* | *p* | *η^2^* | FDR-corrected *p* |
| --- | --- | --- | --- | --- |
| CH8-CH1 | 2.074 | 0.152 | 0.012 | 0.162 |
| CH8-CH2 | 0.690 | 0.407 | 0.004 | 0.407 |
| CH8-CH3 | 2.779 | 0.097 | 0.016 | 0.119 |
| CH8-CH4 | 2.402 | 0.123 | 0.014 | 0.134 |
| CH8-CH5 | 3.002 | 0.085 | 0.017 | 0.110 |
| CH8-CH6 | 3.507 | 0.063 | 0.020 | 0.090 |
| **CH8-CH7*** | **6.049** | **0.015** | **0.034** | **0.028** |
|  |  |  |  |  |
| **CH9-CH1**** | **8.711** | **0.004** | **0.048** | **0.009** |
| **CH9-CH2**** | **9.056** | **0.003** | **0.049** | **0.009** |
| **CH9-CH3***** | **13.544** | **3.11×10^-4^** | **0.072** | **0.003** |
| **CH9-CH4***** | **14.030** | **2.44×10^-4^** | **0.075** | **0.003** |
| **CH9-CH5*** | **5.785** | **0.017** | **0.032** | **0.030** |
| **CH9-CH6**** | **11.090** | **0.001** | **0.060** | **0.006** |
| **CH9-CH7**** | **8.711** | **0.004** | **0.048** | **0.009** |
|  |  |  |  |  |
| CH10-CH1 | 2.580 | 0.110 | 0.015 | 0.125 |
| CH10-CH2 | 2.551 | 0.112 | 0.014 | 0.125 |
| CH10-CH3 | 1.103 | 0.295 | 0.006 | 0.308 |
| CH10-CH4 | 3.306 | 0.071 | 0.019 | 0.098 |
| **CH10-CH5**** | **7.579** | **0.007** | **0.042** | **0.015** |
| CH10-CH6 | 0.714 | 0.399 | 0.004 | 0.407 |
| CH10-CH7 | 4.592 | 0.034 | 0.026 | 0.055 |
|  |  |  |  |  |
| **CH11-CH1**** | **9.845** | **0.002** | **0.054** | **0.009** |
| **CH11-CH2**** | **9.125** | **0.003** | **0.050** | **0.009** |
| **CH11-CH3***** | **13.563** | **3.08×10^-4^** | **0.072** | **0.003** |
| **CH11-CH4***** | **11.415** | **0.001** | **0.062** | **0.006** |
| **CH11-CH5**** | **9.318** | **0.003** | **0.051** | **0.009** |
| **CH11-CH6**** | **10.921** | **0.001** | **0.059** | **0.006** |
| **CH11-CH7***** | **11.941** | **0.001** | **0.064** | **0.005** |
|  |  |  |  |  |
| CH12-CH1 | 3.951 | 0.048 | 0.022 | 0.072 |
| CH12-CH2 | 3.277 | 0.072 | 0.018 | 0.098 |
| **CH12-CH3***** | **14.700** | **1.76×10^-4^** | **0.078** | **0.003** |
| **CH12-CH4***** | **12.369** | **0.001** | **0.066** | **0.005** |
| CH12-CH5 | 3.205 | 0.075 | 0.018 | 0.100 |
| **CH12-CH6***** | **14.609** | **1.84×10^-4^** | **0.077** | **0.003** |
| **CH12-CH7**** | **7.630** | **0.006** | **0.042** | **0.015** |
|  |  |  |  |  |
| CH13-CH1 | 4.234 | 0.041 | 0.024 | 0.065 |
| CH13-CH2 | 2.724 | 0.101 | 0.015 | 0.120 |
| CH13-CH3 | 4.134 | 0.044 | 0.023 | 0.067 |
| **CH13-CH4*** | **5.968** | **0.016** | **0.033** | **0.028** |
| **CH13-CH5*** | **6.769** | **0.010** | **0.037** | **0.021** |
| CH13-CH6 | 2.670 | 0.104 | 0.015 | 0.121 |
| **CH13-CH7*** | **6.425** | **0.012** | **0.036** | **0.025** |
|  |  |  |  |  |
| **CH14-CH1*** | **6.051** | **0.015** | **0.034** | **0.028** |
| CH14-CH2 | 2.851 | 0.093 | 0.016 | 0.117 |
| **CH14-CH3**** | **9.282** | **0.003** | **0.051** | **0.009** |
| **CH14-CH4**** | **9.117** | **0.003** | **0.050** | **0.009** |
| CH14-CH5 | 4.807 | 0.030 | 0.027 | 0.050 |
| **CH14-CH6**** | **9.627** | **0.002** | **0.052** | **0.009** |
| **CH14-CH7**** | **7.429** | **0.007** | **0.041** | **0.016** |

Note: ***** *p* < 0.05, ****** *p* < 0.01, ******* *p* < 0.001, FDR corrected.
