# Supplementary material for: Social bonding in groups of humans selectively increases inter-status information exchange and prefrontal neural synchronization
Source: PLoS Biol. 2024 Mar 19;22(3):e3002545. doi: 10.1371/journal.pbio.3002545 (PMC10950240; doi:10.1371/journal.pbio.3002545)
Supplement: S8 Table — (DOCX) [file pbio.3002545.s020.docx]

**S8 Table. The anatomical position for each recording channel.**

| Channel | MNI coordinates | | | | BA | Brain Regions |
| --- | --- | --- | --- | --- | --- | --- |
|  | *x* | *y* | *z* | *SD* |  |  |
| ***TPJ*** |  |  |  |  |  |  |
| 1 | 59 | -51 | 48 | 11.40 | BA40 | Inferior Parietal Lobe |
| 2 | 55 | -68 | 35 | 11.95 | BA39 | Temporal Parietal Junction |
| 3 | 67 | -43 | 35 | 11.38 | BA40 | Supramarginal gyrus |
| 4 | 64 | -58 | 20 | 11.96 | BA22 | Superior Temporal Gyrus |
| 5 | 57 | -73 | 6 | 12.18 | BA37 | Middle Temporal Gyrus |
| 6 | 69 | -48 | 4 | 11.51 | BA22 | Middle Temporal Gyrus |
| 7 | 62 | -62 | -10 | 13.65 | BA37 | Inferior Temporal Gyrus |
| ***DLPFC*** |  |  |  |  |  |  |
| 8 | 49 | 37 | 34 | 7.41 | BA45 | Middle Frontal Gyrus |
| 9 | 29 | 48 | 43 | 6.77 | BA9 | Dorsolateral Prefrontal Cortex |
| 10 | 55 | 38 | 18 | 7.51 | BA45 | Inferior Frontal Gyrus |
| 11 | 40 | 53 | 28 | 6.81 | BA46 | Dorsolateral Prefrontal Cortex |
| 12 | 18 | 60 | 36 | 6.23 | BA9 | Dorsolateral Prefrontal Cortex |
| 13 | 47 | 54 | 12 | 7.40 | BA46 | Dorsolateral prefrontal cortex |
| 14 | 27 | 65 | 22 | 6.78 | BA10 | Dorsolateral Prefrontal Cortex |
