# Supplementary material for: Social bonding in groups of humans selectively increases inter-status information exchange and prefrontal neural synchronization
Source: PLoS Biol. 2024 Mar 19;22(3):e3002545. doi: 10.1371/journal.pbio.3002545 (PMC10950240; doi:10.1371/journal.pbio.3002545)
Supplement: S9 Table — (DOCX) [file pbio.3002545.s021.docx]

**S9 Table. Full statistical reports of the results of Hierarchy × Bonding mixed-model ANOVAs on inter-brain neural synchronization in frequency band 0.407-0.432Hz.**

| Channel | Effect | *F* | *p* | *η^2^* | FDR-corrected *p* |
| --- | --- | --- | --- | --- | --- |
| ***TPJ*** |  |  |  |  |  |
| 1 | Bonding | 0.006 | 0.936 | 3.66×10^-5^ | 0.952 |
|  | Hierarchy | 1.001 | 0.318 | 0.006 | 0.988 |
|  | Bonding ×Hierarchy | 0.388 | 0.534 | 0.002 | 0.831 |
|  |  |  |  |  |  |
| 2 | Bonding | 0.321 | 0.572 | 0.002 | 0.952 |
|  | Hierarchy | 0.095 | 0.758 | 0.001 | 0.988 |
|  | Bonding ×Hierarchy | 2.288 | 0.132 | 0.013 | 0.831 |
|  |  |  |  |  |  |
| 3 | Bonding | 0.067 | 0.796 | 3.85×10^-4^ | 0.952 |
|  | Hierarchy | 0.298 | 0.586 | 0.002 | 0.988 |
|  | Bonding ×Hierarchy | 0.017 | 0.897 | 9.64×10^-5^ | 0.897 |
|  |  |  |  |  |  |
| 4 | Bonding | 3.896 | 0.050 | 0.022 | 0.350 |
|  | Hierarchy | 0.933 | 0.336 | 0.005 | 0.988 |
|  | Bonding ×Hierarchy | 1.944 | 0.165 | 0.011 | 0.831 |
|  |  |  |  |  |  |
| 5 | Bonding | 0.145 | 0.703 | 0.001 | 0.952 |
|  | Hierarchy | 0.024 | 0.878 | 1.35×10^-4^ | 0.988 |
|  | Bonding ×Hierarchy | 1.780 | 0.184 | 0.010 | 0.831 |
|  |  |  |  |  |  |
| 6 | Bonding | 0.024 | 0.877 | 1.38×10^-4^ | 0.952 |
|  | Hierarchy | 0.010 | 0.919 | 5.95×10^-5^ | 0.988 |
|  | Bonding ×Hierarchy | 0.445 | 0.505 | 0.003 | 0.831 |
|  |  |  |  |  |  |
| 7 | Bonding | 1.095 | 0.297 | 0.006 | 0.820 |
|  | Hierarchy | 0.080 | 0.778 | 4.57×10^-4^ | 0.988 |
|  | Bonding ×Hierarchy | 0.205 | 0.652 | 0.001 | 0.897 |
| ***DLPFC*** |  |  |  |  |  |
| 8 | Bonding | 2.361 | 0.126 | 0.013 | 0.589 |
|  | Hierarchy | 2.11×10^-4^ | 0.988 | 1.12×10^-6^ | 0.988 |
|  | Bonding ×Hierarchy | 0.603 | 0.439 | 0.003 | 0.831 |
|  |  |  |  |  |  |
| 9 | Bonding | 3.989 | 0.047 | 0.022 | 0.350 |
|  | Hierarchy | 3.232 | 0.074 | 0.018 | 0.988 |
|  | Bonding ×Hierarchy | 0.483 | 0.488 | 0.003 | 0.831 |
|  |  |  |  |  |  |
| 10 | Bonding | 0.343 | 0.559 | 0.002 | 0.988 |
|  | Hierarchy | 0.067 | 0.797 | 3.83×10^-4^ | 0.897 |
|  | Bonding ×Hierarchy | 1.504 | 0.222 | 0.009 | 0.776 |
|  |  |  |  |  |  |
| 11 | Bonding | 0.146 | 0.703 | 0.001 | 0.952 |
|  | Hierarchy | 0.164 | 0.686 | 0.001 | 0.988 |
|  | Bonding ×Hierarchy | 0.053 | 0.818 | 3.04×10^-4^ | 0.897 |
|  |  |  |  |  |  |
| 12 | Bonding | 0.873 | 0.351 | 0.005 | 0.820 |
|  | Hierarchy | 0.081 | 0.777 | 4.64×10^-4^ | 0.988 |
|  | Bonding ×Hierarchy | 0.403 | 0.526 | 0.002 | 0.831 |
|  |  |  |  |  |  |
| 13 | Bonding | 0.004 | 0.952 | 2.65×10^-5^ | 0.952 |
|  | Hierarchy | 1.628 | 0.204 | 0.009 | 0.988 |
|  | Bonding ×Hierarchy | 0.045 | 0.833 | 2.57×10^-4^ | 0.897 |
|  |  |  |  |  |  |
| 14 | Bonding | 0.415 | 0.520 | 0.002 | 0.952 |
|  | Hierarchy | 0.104 | 0.748 | 0.001 | 0.988 |
|  | Bonding ×Hierarchy | 0.471 | 0.493 | 0.003 | 0.831 |
