# Supplementary material for: Social bonding in groups of humans selectively increases inter-status information exchange and prefrontal neural synchronization
Source: PLoS Biol. 2024 Mar 19;22(3):e3002545. doi: 10.1371/journal.pbio.3002545 (PMC10950240; doi:10.1371/journal.pbio.3002545)
Supplement: S10 Table — (A) Analysis on pre- and post-leader emergence stages (based on leader emergence time). (B) Analysis on early and late time-bin (equally split by mid-point). (DOCX) [file pbio.3002545.s022.docx]

**S10 Table. Statistical reports of interaction-stage/time-bin modulation effects on main findings.**

| Indices in within-group interaction | Examination | Effect | *F* | *p* | *η^2^* |
| --- | --- | --- | --- | --- | --- |
| ***Behavioral*** |  |  |  |  |  |
| Turn transition | **Observe effect**** | **hierarchy× bonding** | **8.395** | **0.004** | **0.046** |
|  | Stage modulating effect | stage × hierarchy× bonding | 0.057 | 0.812 | 3.27×10^-4^ |
| Turn response time | **Observe effect***** | **bonding** | **17.511** | **5.10×10^-5^** | **0.115** |
|  | Stage modulating effect | stage × bonding | 0.134 | 0.715 | 0.001 |
| ***Neural*** |  |  |  |  |  |
| INS in rTPJ (CH3) | **Observe effect**** | **hierarchy** | **10.585** | **0.001** | **0.057** |
|  | Stage modulating effect | stage × hierarchy | 0.529 | 0.468 | 0.003 |
| INS in rDLPFC (CH9) | **Observe effect**** | **hierarchy× bonding** | **11.312** | **0.001** | **0.061** |
|  | Stage modulating effect | stage × hierarchy× bonding | 3.064 | 0.082 | 0.017 |

**(A****).** Analysis on pre- and post-leader emergence stages

**(B).** Analysis on early and late time-bin (equally split by mid-point).

Note: ***** *p* < 0.05, ****** *p* < 0.01, ******* *p* < 0.001.

| Indices in within-group interaction | Examination | Effect | *F* | *p* | *η^2^* |
| --- | --- | --- | --- | --- | --- |
| ***Behavioral*** |  |  |  |  |  |
| Turn transition | **Observe effect**** | **hierarchy× bonding** | **11.246** | **0.001** | **0.061** |
|  | Stage modulating effect | stage × hierarchy× bonding | 0.064 | 0.800 | 3.71×10^-4^ |
| Turn response time | **Observe effect**** | **bonding** | **10.852** | **0.001** | **0.074** |
|  | Stage modulating effect | stage × bonding | 2.538 | 0.113 | 0.018 |
| ***Neural*** |  |  |  |  |  |
| INS in rTPJ (CH3) | **Observe effect**** | **hierarchy** | **10.204** | **0.002** | **0.055** |
|  | Stage modulating effect | stage × hierarchy | 0.011 | 0.917 | 6.30×10^-6^ |
| INS in rDLPFC (CH9) | **Observe effect**** | **hierarchy× bonding** | **9.280** | **0.003** | **0.051** |
|  | Stage modulating effect | stage × hierarchy× bonding | 2.168 | 0.143 | 0.012 |
