# Supplementary material for: Social bonding in groups of humans selectively increases inter-status information exchange and prefrontal neural synchronization
Source: PLoS Biol. 2024 Mar 19;22(3):e3002545. doi: 10.1371/journal.pbio.3002545 (PMC10950240; doi:10.1371/journal.pbio.3002545)
Supplement: S11 Table — (DOCX) [file pbio.3002545.s023.docx]

**S11 Table. Full statistical reports of the results of Hierarchy × Bonding mixed-model ANOVAs on HbR-INS.**

| Channel | Effect | *F* | *p* | *η^2^* | FDR-corrected *p* |
| --- | --- | --- | --- | --- | --- |
| ***TPJ*** |  |  |  |  |  |
| 1 | Bonding | 0.001 | 0.978 | 4.51×10^-6^ | 0.978 |
|  | Hierarchy | 0.365 | 0.546 | 0.002 | 0.846 |
|  | Bonding × Hierarchy | 2.721 | 0.101 | 0.015 | 0.785 |
|  |  |  |  |  |  |
| 2 | Bonding | 3.792 | 0.053 | 0.021 | 0.372 |
|  | Hierarchy | 0.137 | 0.712 | 0.001 | 0.846 |
|  | Bonding × Hierarchy | 1.041 | 0.309 | 0.006 | 0.785 |
|  |  |  |  |  |  |
| 3 | Bonding | 0.165 | 0.685 | 0.001 | 0.978 |
|  | Hierarchy | 4.736 | 0.031 | 0.026 | 0.432 |
|  | Bonding × Hierarchy | 0.130 | 0.719 | 0.001 | 0.785 |
|  |  |  |  |  |  |
| 4 | Bonding | 0.103 | 0.748 | 0.001 | 0.978 |
|  | Hierarchy | 0.170 | 0.680 | 0.001 | 0.846 |
|  | Bonding × Hierarchy | 0.760 | 0.384 | 0.004 | 0.785 |
|  |  |  |  |  |  |
| 5 | Bonding | 3.886 | 0.050 | 0.022 | 0.372 |
|  | Hierarchy | 0.123 | 0.726 | 0.001 | 0.846 |
|  | Bonding × Hierarchy | 0.123 | 0.726 | 0.001 | 0.785 |
|  |  |  |  |  |  |
| 6 | Bonding | 0.015 | 0.903 | 8.63×10^-5^ | 0.978 |
|  | Hierarchy | 0.475 | 0.492 | 0.003 | 0.846 |
|  | Bonding × Hierarchy | 0.670 | 0.414 | 0.004 | 0.785 |
|  |  |  |  |  |  |
| 7 | Bonding | 0.040 | 0.842 | 2.30×10^-4^ | 0.978 |
|  | Hierarchy | 2.855 | 0.093 | 0.016 | 0.650 |
|  | Bonding × Hierarchy | 0.691 | 0.407 | 0.004 | 0.785 |
| ***DLPFC*** |  |  |  |  |  |
| 8 | Bonding | 0.718 | 0.398 | 0.004 | 0.978 |
|  | Hierarchy | 0.304 | 0.582 | 0.002 | 0.846 |
|  | Bonding × Hierarchy | 0.383 | 0.537 | 0.002 | 0.785 |
|  |  |  |  |  |  |
| 9 | Bonding | 0.169 | 0.682 | 0.001 | 0.978 |
|  | Hierarchy | 0.038 | 0.846 | 2.17×10^-4^ | 0.846 |
|  | Bonding × Hierarchy | 0.420 | 0.518 | 0.002 | 0.785 |
|  |  |  |  |  |  |
| 10 | Bonding | 0.160 | 0.689 | 0.001 | 0.978 |
|  | Hierarchy | 0.314 | 0.576 | 0.002 | 0.846 |
|  | Bonding × Hierarchy | 0.923 | 0.338 | 0.005 | 0.785 |
|  |  |  |  |  |  |
| 11 | Bonding | 0.179 | 0.673 | 0.001 | 0.978 |
|  | Hierarchy | 1.847 | 0.176 | 0.011 | 0.821 |
|  | Bonding × Hierarchy | 0.115 | 0.734 | 0.001 | 0.785 |
|  |  |  |  |  |  |
| 12 | Bonding | 0.009 | 0.924 | 5.26×10^-5^ | 0.978 |
|  | Hierarchy | 0.546 | 0.461 | 0.003 | 0.846 |
|  | Bonding × Hierarchy | 0.074 | 0.785 | 4.27×10^-4^ | 0.785 |
|  |  |  |  |  |  |
| 13 | Bonding | 0.112 | 0.739 | 0.001 | 0.978 |
|  | Hierarchy | 0.074 | 0.785 | 4.28×10^-4^ | 0.846 |
|  | Bonding × Hierarchy | 0.353 | 0.553 | 0.002 | 0.785 |
|  |  |  |  |  |  |
| 14 | Bonding | 1.241 | 0.267 | 0.007 | 0.978 |
|  | Hierarchy | 0.116 | 0.733 | 0.001 | 0.846 |
|  | Bonding × Hierarchy | 0.079 | 0.779 | 4.53×10^-4^ | 0.785 |

Note: FDR corrected for 14 channels.
