# Supplementary material for: Social bonding in groups of humans selectively increases inter-status information exchange and prefrontal neural synchronization
Source: PLoS Biol. 2024 Mar 19;22(3):e3002545. doi: 10.1371/journal.pbio.3002545 (PMC10950240; doi:10.1371/journal.pbio.3002545)
Supplement: S12 Table — (DOCX) [file pbio.3002545.s024.docx]

**S12 Table. Full statistical reports of hierarchy main effect on rDLPFC-rTPJ HbR-FC for each channel pair.**

| Channel pairs  (rDLPFC-rTPJ) | *F* | *p* | *η^2^* | FDR-corrected *p* |
| --- | --- | --- | --- | --- |
| **CH8-CH1**** | **8.567** | **0.004** | **0.047** | **0.005** |
| **CH8-CH2*** | **5.587** | **0.019** | **0.031** | **0.021** |
| **CH8-CH3**** | **9.718** | **0.002** | **0.053** | **0.003** |
| **CH8-CH4*** | **4.450** | **0.036** | **0.025** | **0.037** |
| **CH8-CH5**** | **10.234** | **0.002** | **0.056** | **0.003** |
| **CH8-CH6***** | **13.449** | **3.26×10^-4^** | **0.072** | **0.001** |
| **CH8-CH7***** | **18.929** | **2.31×10^-5^** | **0.098** | **1.03×10^-4^** |
|  |  |  |  |  |
| **CH9-CH1**** | **7.953** | **0.005** | **0.044** | **0.007** |
| **CH9-CH2***** | **13.787** | **2.75×10^-4^** | **0.073** | **0.001** |
| **CH9-CH3***** | **20.285** | **1.22×10^-5^** | **0.104** | **6.93×10^-5^** |
| **CH9-CH4***** | **16.761** | **6.48×10^-5^** | **0.088** | **2.27×10^-4^** |
| **CH9-CH5***** | **14.131** | **2.33×10^-4^** | **0.075** | **0.001** |
| **CH9-CH6***** | **23.947** | **2.25×10^-6^** | **0.121** | **2.75×10^-5^** |
| **CH9-CH7**** | **7.953** | **0.005** | **0.044** | **0.007** |
|  |  |  |  |  |
| **CH10-CH1**** | **11.738** | **0.001** | **0.063** | **0.001** |
| **CH10-CH2*** | **6.676** | **0.011** | **0.037** | **0.013** |
| **CH10-CH3*** | **4.970** | **0.027** | **0.028** | **0.029** |
| CH10-CH4 | 1.328 | 0.251 | 0.008 | 0.251 |
| **CH10-CH5**** | **9.556** | **0.002** | **0.052** | **0.003** |
| **CH10-CH6*** | **5.443** | **0.021** | **0.030** | **0.023** |
| **CH10-CH7***** | **13.978** | **2.51×10^-4^** | **0.074** | **0.001** |
|  |  |  |  |  |
| **CH11-CH1**** | **9.693** | **0.002** | **0.053** | **0.003** |
| **CH11-CH2**** | **9.359** | **0.003** | **0.051** | **0.003** |
| **CH11-CH3***** | **29.023** | **2.30×10^-7^** | **0.143** | **1.13×10^-5^** |
| **CH11-CH4***** | **13.983** | **2.50×10^-4^** | **0.074** | **0.001** |
| **CH11-CH5**** | **12.300** | **0.001** | **0.066** | **0.001** |
| **CH11-CH6***** | **25.266** | **1.23×10^-6^** | **0.127** | **2.01×10^-5^** |
| **CH11-CH7***** | **26.450** | **7.23×10^-7^** | **0.132** | **1.77×10^-5^** |
|  |  |  |  |  |
| **CH12-CH1*** | **4.518** | **0.035** | **0.025** | **0.036** |
| **CH12-CH2**** | **7.519** | **0.007** | **0.041** | **0.008** |
| **CH12-CH3***** | **22.629** | **4.11×10^-6^** | **0.115** | **3.77×10^-5^** |
| **CH12-CH4***** | **13.286** | **3.53×10^-4^** | **0.071** | **0.001** |
| **CH12-CH5**** | **10.759** | **0.001** | **0.058** | **0.002** |
| **CH12-CH6***** | **17.970** | **3.64×10^-5^** | **0.094** | **1.37×10^-4^** |
| **CH12-CH7***** | **18.294** | **3.12×10^-5^** | **0.095** | **1.27×10^-4^** |
|  |  |  |  |  |
| **CH13-CH1**** | **10.067** | **0.002** | **0.055** | **0.003** |
| **CH13-CH2*** | **6.615** | **0.011** | **0.037** | **0.013** |
| **CH13-CH3**** | **11.024** | **0.001** | **0.060** | **0.002** |
| **CH13-CH4*** | **5.846** | **0.017** | **0.033** | **0.019** |
| **CH13-CH5**** | **11.479** | **0.001** | **0.062** | **0.002** |
| **CH13-CH6**** | **11.384** | **0.001** | **0.061** | **0.002** |
| **CH13-CH7***** | **21.132** | **8.22×10^-6^** | **0.108** | **5.75×10^-5^** |
|  |  |  |  |  |
| **CH14-CH1***** | **16.494** | **7.37×10^-5^** | **0.087** | **2.41×10^-4^** |
| **CH14-CH2***** | **14.440** | **2.00×10^-4^** | **0.077** | **0.001** |
| **CH14-CH3***** | **22.375** | **4.62×10^-6^** | **0.114** | **3.77×10^-5^** |
| **CH14-CH4***** | **13.209** | **3.67×10^-4^** | **0.071** | **0.001** |
| **CH14-CH5**** | **9.754** | **0.002** | **0.053** | **0.003** |
| **CH14-CH6***** | **19.488** | **1.77×10^-5^** | **0.101** | **8.69×10^-5^** |
| **CH14-CH7***** | **20.193** | **1.21×10^-5^** | **0.104** | **6.93×10^-5^** |

Note: ***** *p* < 0.05, ****** *p* < 0.01, ******* *p* < 0.001, FDR corrected.
