# Supplementary material for: Social bonding in groups of humans selectively increases inter-status information exchange and prefrontal neural synchronization
Source: PLoS Biol. 2024 Mar 19;22(3):e3002545. doi: 10.1371/journal.pbio.3002545 (PMC10950240; doi:10.1371/journal.pbio.3002545)
Supplement: Attachment — Submitted filename: Point-by-point response_revised.docx [file pbio.3002545.s025.docx]

- **Responses to Reviewer #1’s comments:**

The manuscript reports an impressive study on the effects of a social bonding intervention on hierarchical interactions in small groups of three people. The topic is very interesting and timely. A great strength of the study is the large data set that was collected and the analysis of both intra- and inter-status interaction patterns on the behavioral and neural levels.

I have some questions and suggestions to further improve the manuscript:

*Overall response to Reviewer #1’s comment:* We greatly appreciate Reviewer #1’s positive evaluations of our manuscript, as well as the constructive suggestions for improvement. In response, we have implemented the following revisions– both in the point-by-point response below and in the revised manuscript:

**i)** In the revised *Introduction*, we have elaborated on our research questions, key manipulations, and justification of regions of interest. **ii)** We conducted additional analysis and replicated our main findings on hierarchical interaction when we used coherence values for the leader and a randomly selected follower to index inter-status neural synchronization, or applied linear mixed model (LMM) analyses to all statistical testing presented in the main text. We also examined the effects on deoxygenated hemoglobin signals (HbR). **iii)** In the revised *Discussion*, we have elaborated on our understanding of socio-cognitive mechanisms underlying the effect of bonding manipulation on inter-status interaction patterns. We also discussed cultural specificity/generality related to our findings. **iv)** Methodological details for pre-processing and data quality check have been included in the revised *Methods*.

\# Point 1a. The manuscript would benefit from clear theory-driven hypotheses. Did the authors expect to find differential effects of the bonding manipulation on intra- vs. inter-status interactions? If yes, these would be good to delineate. If the study was more explorative in nature, this is fine as well, but it should be made explicit.
\# Point 1b.Relatedly: Was there a pre-registration? If yes, this should be made public and linked to the manuscript. If not, this should be mentioned.

***Response*:** We thank Reviewer #1 for this constructive suggestion. Accordingly, in the revised *Introduction* (page 4, lines 85-96), we have clarified our research question of investigating whether and, if so, how ingroup social bonding differently influenced different types of interpersonal interactions within a hierarchical group and we addressed it at both the behavioral and neural levels. Furthermore, we have developed specific hypotheses regarding the ‘whether’ question. Previous studies have demonstrated distinct functions of inter-status and intra-status interactions within hierarchical groups. The inter-status interaction enables asymmetric information exchange between the group leader and followers (Biro et al., 2006; Couzin et al., 2005), as well as leader-follower coordination and alignment (Podolny & Baron, 1997; Zhang et al., 2019). On the other hand, the intra-status interaction fosters reciprocal relationships and peer support among individuals with similar social status (e.g., followers) (Hüffmeier et al., 2014; Sapolsky, 2005; Ostner et al., 2008). Therefore, we expected that social bonding would exert different effects on inter-status and intra-status interpersonal interactions within a hierarchical group. However, based on existing literature, the direction of this influence was less clear. Regarding the ‘how’ question, we did not formulate specific hypotheses regarding the direction of the interaction; it could be either stronger or weaker or even exhibit opposite effects of social bonding on inter- vs. intra-status interactions. As a result, we consider it an open question that necessitates investigation in our current study.

Regarding the pre-registration question, we clarified that the current study has not been pre-registered in the revised *Materials and Methods* (page 28, lines 719-720).

\# Point 2. Please briefly mention the type of social bonding manipulation that was used in the introduction. I kept wondering, but I did not want to switch back and forth between the sections. A sentence or two will suffice.

***Response*:** We thank Reviewer #1 for asking us to clarify these issues. This should indeed be clear to the reader earlier. Accordingly, we have briefly mentioned how we manipulated in-group social bonding in the revised *Introduction* (page 5, lines 131-135).

page 5, lines 131-135: “The in-group social bonding manipulation employed in the current study was adapted from several previously validated procedures [21,63-66]. Specifically, we integrated three fundamental procedures to manipulate in-group social bonding: i) shared preference [67-68], ii) symbolic marker [64,69], and iii) similarity among group members [65,70].”

\# Point 3. Why were fNIRS recordings only performed over participants' right hemispheres? A clearer rationale would be helpful.

***Response:*** We appreciate the suggestion from Reviewer #1 and have accordingly specified in the revised *Introduction* (pages 5-6, lines 139-149) the hemisphere information of previous findings. Furthermore, we have clarified that our selection of the right hemisphere’s DLPFC and TPJ as regions of interest was motivated by previous research indicating their involvement in social decision-making and group interaction. Specifically, it has been shown that the right (but not) DLPFC was engaged in allocating attention and making strategic decision during social interaction (Zink et al., 2008; Jeurissen et al., 2014; Rilling & Sanfey, 2011). The TPJ, particularly in the right hemisphere, has been associated with understanding others’ mental states (Young et al., 2010), as well as alignment with in-group members regarding consensus decision and group norms (Bitsch et al., 2018; Saxe & Wexler, 2005). Our choice was also based on earlier studies that have linked neural synchronization in the right DLPFC and right TPJ to social interactive processes (Cui et al., 2012; Lu et al., 2019; Xie et al., 2020) and ingroup cooperation (Yang et al., 2020, Zhou et al., 2022), while highlighting the role of neural synchronization in the right TPJ in leader-follower interaction (Liang et al., 2022; He et al., 2023).

\# Point 4. A figure outlining the sequence of tasks and measures would be helpful for the reader.

***Response*:** We thank Reviewer #1 for this constructive suggestion. Accordingly, we provided a figure to illustrate the experimental procedure in the revision (Fig R1; S1 Fig).

**Fig. R1.** **Experimental procedure.** Before coming to the laboratory, participants completed an online survey that included demographic and psychological information as well as color preference. One to four days later, participants were invited to the laboratory in groups of three same-gender strangers and randomly assigned into either the bonding or control condition. They were instructed to sit face-to-face in a triangle and completed 3 sessions during fNIRS-based hyper-scanning: i) a 4-min resting-state session; ii) a 4-min ingroup social bonding (or no-bonding control) manipulation session; and iii) a 4-min online within-group interaction session. At the end of the experiment, participants were asked to complete a series of intergroup-related economic games (including intergroup dictator game and intergroup prisoner’s dilemma-maximizing differences game), as well as report subjective evaluations on group cohesion, leader influence and attraction, positive attitudes, willingness to become the leader, etc. (details in *Methods*).

\# Point 5. The authors report only HbO data and try to justify their choice in the methods section. Yet, it is good practice to also report HbR data in fNIRS research. They should add corresponding analyses, at the very least as additional analyses in the supplements. If the same patterns are found as with HbO, that's great. If no significant results can be found that is also informative for other researchers and it could support the authors' notion that HbO is the more sensitive measure.

***Response*:** We appreciate this suggestion. To address this point, we repeated our analysis on the deoxygenated hemoglobin signals (HbR). Specifically, we calculated the inter-brain neural synchronization (INS) and intra-brain rDLPFC-rTPJ functional connectivity (FC) on HbR signals. We then performed the bonding × hierarchy ANOVAs on HbR-INS and HbR-FC to examine whether the main findings obtained with HbO signals would be similarly observed with HbR signals. We reported the results of HbR signals below (Fig R2) as well as in the revision (S11 Fig). Basically, the analysis on the HbR signals showed **i)** a much weaker effect on HbR-INS than that of HbO signals (Fig R2A), but **ii)** similar effects on the intra-brain FC based on HbO and HbR signals (Fig R2B).

Regarding the INS index, some of previous studies found similar but weaker effects based on HbR and HbO signals (Pan et al., 2017; Yang et al., 2020), whereas other studies only found a significant effect on INS based on HbO, but not HbR, signals (Liu et al., 2019; Vanzella et al., 2019). In the current study, similar to the pattern of the HbO signals, we observed a significant, although weaker (did not survive multiple corrections), hierarchy effect in rTPJ (channel 3, *F*_1, 174_ = 4.736, *uncorrected* *p* = 0.031, *η^2^* = 0.026, Table R1A, S11 Table; Fig R2A, S11A Fig), with stronger inter-status INS than intra-status dyads. However, no bonding × hierarchy interaction effect was observed on HbR signals (channel 9, *F*_1, 174_ = 0.420, *p* = 0.518, *η^2^* = 0.002, Table R1A, S11 Table). Such similarity and discrepancy in HbR and HbO signals were consistent with previous observations (Pan et al., 2017; Yang et al., 2020; Liu et al., 2019; Vanzella et al., 2019).

Regarding the intra-brain FC index, the leader effect on the rDLPFC-rTPJ functional connectivity (stronger in leader than followers) based on HbO was similarly observed in the HbR-FC analysis (48 rDLPFC-rTPJ channel pairs survived FDR correction for 49 channel pairs, Table R1B, S12 Table; Fig R2B, S11B Fig for the grand mean rDLPFC-rTPJ connectivity, two way mixed-model ANOVA, *F*_1, 174_ = 27.345, *p* = 4.842 × 10^-7^, *η^2^* = 0.136).

The difference observed on INS based on HbO and HbR signals may be caused by different sensitivities of these two types of signals in reflecting task-induced changes in neural signals. Moreover, previous studies have shown that the HbO signal has a higher signal-to-noise ratio than the HbR signal (Ding et al., 2014; Homae et al., 2007; Taga et al., 2003; Zhang et al., 2018) and is a more sensitive indicator of changes in regional cerebral blood flow (Hoshi, 2007; Cui et al., 2011; Tachtsidis & Scholkmann, 2016), whereas the HbR signal is more related to neurovascular coupling (Ou et al., 2009). Therefore, we reported the results based on the HbO signals in the main text. To present the measured brain activity from different perspectives, the results based on HbR signals were also reported in the revised *Method* (pages 42-43, lines 1153-1169) and *SI* **(**S11-S12 Table, S11 Fig).

**Fig R2. Inter-brain neural synchronization and intra-brain functional connectivity in HbR signals.** (**A**) HbR-INS in rTPJ (channel 3) showed a significant, but weaker (did not survive multiple corrections), hierarchy main effect, with stronger inter-status INS than intra-status one. (**B**) The grand mean of rDLPFC-rTPJ connectivity in HbR exhibited a significant hierarchy main effect, with stronger rDLPFC-rTPJ functional connectivity in leaders (vs. followers). ***p < 0.001.

\# Point 6. Permutation analysis: Maybe I missed this, but did the authors find significantly increased WTC in real groups vs. random groups? That should be stated.

***Response:*** This is a very valid point, thanks. Indeed, in the original submission, we focused on the effects of bonding and/or hierarchy on the WTC values (i.e., main effect of hierarchy on the rTPJ INS, Fig 3B and bonding × hierarchy interaction on the rDLPFC INS, Fig 3D) and validated the observed effects with permutation tests (Fig 3F, G). Following this suggestion, we conducted corresponding permutation analyses and confirmed increased inter-brain neural synchronization (INS) in real groups compared to pseudo groups. The details in these analyses and results were reported below and in the revised *Methods* (pages 39-40, lines 1068-1090).

To address this issue, we first examined which conditions showed increased INS (i. e., significant INS increases during group interaction compared to resting-state). Specifically, we compared INS differences (group interaction minus resting-state) against zero for the channels that exhibited significant effects of interest (i.e., channel 3 in the rTPJ and channel 9 in the rDLPFC). We found increased INS for the inter-status dyads at channel 3 in the rTPJ under control condition (*t*_1,86_ = 3.943, *p* = 1.64×10^-4^) and bonding condition (*t*_1,88_ = 3.394, *p* = 0.001; survived multiple correction; Table R2) and at channel 9 in the rDLPFC (bonding: *t*_1,88_ = 3.378, *p* = 0.001; survived multiple correction; Table R2). Next, we performed permutation test to examine whether these conditions showed increased INS in real group than pseudo groups.

Specifically, within each condition, we generated 3-person pseudo-groups by randomly grouping 3 participants from different original real groups. For each pseudo-group, the INS of each dyad was recalculated. This procedure was repeated for 1000 times to generate a pseudo-group INS distribution. The increased INS in the aforementioned conditions of real groups were compared with each condition-specific permutation distributions. The results indicated that the increases in inter-status INS in the rTPJ (under control condition: *p* = 0.022; bonding: *p* = 0.047; Table R2) and rDLPFC (under bonding condition: *p* = 0.032; Table R2) of real groups exceeded the upper 95% *CI* of the permutation distribution. These findings further confirmed an increased INS in these specific conditions within real (rather than random) groups.

\# Point 7. In the discussion, I would be interested to learn more about the authors' thoughts on the underlying socio-cognitive mechanisms of how exactly the bonding manipulation influenced inter-status interaction patterns, in particular.

***Response:*** We thank Reviewer #1 for this constructive suggestion. Accordingly, in the revised *Discussion*, we have now provided a comprehensive understanding of the impacts of social bonding on inter-status interaction by addressing the following aspects: i) elucidating how social bonding influenced behavioral interaction patterns between group leader and followers (page 23, lines 572-589); ii) explaining how social bonding increased neural alignment between leader and followers (pages 24-25, lines 591-622); and iii) establishing the link between neural effects and behavioral observations (pages 25-26, lines 650-662). We proposed potential socio-cognitive mechanisms underlying the effects of social bonding on inter-status interaction patterns.

page 23, lines 572-589: “By differentiating between inter-status and intra-status interactions in hierarchical groups, we are able to arbitrate between two possible mechanisms: whether social bonding enhances group dynamics in hierarchical groups regardless of the interaction types (i.e., enhancing both inter-status and intra-status interactions in a similar way), or if it is modulated by the interaction type, selectively promoting either inter- or intra-status interaction ... Unlike non-hierarchical groups in which social bonding fosters interaction and cohesion among group members indiscriminately, social bonding in hierarchical groups primarily serves to foster and reinforce connections among individuals with different social statuses. It selectively enhances mutual understanding and information exchange between leaders and followers rather than among peers, highlighting the importance of status-related interactions in shaping the effects of social bonding within a hierarchical group.”

pages 24-25, lines 591-622: “We further investigated how social bonding facilitated neural entrainment between individuals of high and low status, specifically addressing whether this entrainment occurred in a high-to-low status or low-to-high status direction or both (i.e., a bidirectional manner). ... Previous studies have associated such sequential neural alignment with anticipatory processing, reflecting active engagement and predictive processing of others’ behaviors and intentions during social interactions [87,104]. For example, in dyadic communication, the listener’s prefrontal activity often precedes that of the speaker, and such anticipatory neural response facilitated mutual understanding and successful communication [94]. Therefore, our findings of leader-to-follower neural alignment suggested a potential underlying process through which social bonding influenced inter-status interaction: the group leader actively engaged in anticipating and predicting the mental states of followers, enabling more frequent communication and coordination with them. Moreover, the effects of social bonding on synchronized and leader-proceeding neural entrainment were only observed in the rDLPFC rather than the rTPJ. Previous work has evidenced a crucial role of the prefrontal cortex, particularly the DLFPC, in predicting forthcoming sensory inputs (e.g., short sound, [105]), social cues (e.g., eye gaze, [90]), and social dominance and future social interactions [45], suggesting DLPFC as a hub region for monitoring errors and updating predictions of upcoming inputs to prepare for appropriate responses [105]. Therefore, the impact of social bonding on rDLPFC synchronization and leader-to-follower alignment may provide a neurocognitive account for increased leader initiation and more frequent, efficient leader-follower information exchange. Interestingly, we showed that group leaders with stronger DLPFC-TPJ functional connectivity exhibited a greater degree of neural alignment with followers. This link suggested that the exchange and integration of information between DLPFC and TPJ may support predictive neural alignment from leader to follower. Taken together, in-group social bonding may enable the group leader to actively adopt followers’ perspective, consider their potential behaviors and intentions, and align predictively with them.”

pages 25-26, lines 650-662: “our work reveals the critical roles of social status in shaping the strength and nature of the social bonding experience in hierarchical groups, which operates at both the individual and dyadic levels. At the individual level, social bonding facilitates the initiation and engagement of high-status individuals in group communications, while increases the responsiveness of low-status individuals to group leader and their perceptions of high-status individuals. At the dyadic level, social bonding exerts distinct effects on the inter-status and intra-status dynamics, potentially through the mechanisms of top-down predictive alignment and bottom-up attentional shift. Specifically, social bonding increases a leader’s forward-prediction of follower’s neural activity, while suppresses follower’s neural and attentional entrainment with same-status fellows. These plausible neurocognitive pathways helped us to synthesize social bonding effects, providing neural accounts for the effect of social bonding on group dynamics within hierarchical social contexts.”

\# Point 8. Conversation analysis: Did participants see when another person was typing? Was this indicated in the chat window? I would imagine that this has quite a strong effect on the response latencies as - different from vocal conversation - it is in principle possible to formulate a response simultaneously in written conversation.

***Response:*** We fully agree with Reviewer #1’s concern regarding the potential impact of displaying “typing” in the group chat window on response latency measurement. To mitigate this potential influence, we have opted not to show status information (i.e., whether someone is typing or not) in the chat window. Only after participants have completed and submitted their messages into the group chat window, other members are then able to read others’ messages. These specific details are important for readers to understand our measurement and we have included them in the revised *Methods* section (page 33, lines 880-884).

\# Point 9. INS: Analysis: There was twice as much data included in the WTC for inter-status than intra-status couples. Was this taken into consideration in the statistical analysis?

***Response:*** This is an excellent point, and we now have conducted a series of control analyses to address such differences when calculating INS for inter-status and intra-status dyads. The results from these analyses, which ensured the same data size for inter- and intra-status dyads, fully replicated our previous findings (Fig 3B, D; S10 Fig; Fig R3).

Specifically, in our initial analysis on INS, we averaged the coherence values of two leader-follower dyads to index inter-status INS, while using one follower-follower dyad per group to index intra-status INS. In response to the reviewer’s inquiry, we modified our calculation method in the control analyses by calculating coherence values for the leader and a randomly selected follower to index inter-status INS. This adjustment allowed us to ensure that the calculation of inter- and intra-status INS was matched. The results from this set of analyses completely reproduced our main findings, including the hierarchy main effect in channel 3 (*F*_1, 174_ = 8.330, *p* = 0.004, *η^2^* = 0.046, Fig R3A, S10A Fig), the bonding × hierarchy interaction effect in channel 9 (*F*_1, 174_ = 11.133, *p* = 0.001, *η^2^* = 0.060, Fig R3B, S10B Fig).

Details regarding these methodological adjustments and their corresponding results were provided in the revised *Methods* (page 37, lines 996-1000) as well as Supplementary Figures (S10 Fig).

**Fig R3. Neural synchronization between the group leader and a randomly selected follower.** The significant hierarchy main effect in the rTPJ (**A**) and bonding × hierarchy interaction effect in the rDLPFC (**B**) were fully replicated when considering inter-status INS for the leader and a randomly selected follower. ***p* < 0.01.

\# Point 10. Information on the fNIRS pre-processing seems to be missing. Please explain your pre-processing steps, including motion correction, data quality checks, exclusion of noisy channels, filtering etc.

***Response:*** We thank Reviewer #1 for this reminder. Accordingly, we have provided details regarding the fNIRS pre-processing in the revised *Methods* (page 36, lines 953-977; pages 37-38, lines 1002-1029).

page 36, lines 953-977: “*Data quality check.* The following steps were performed to check and ensure the data quality of fNIRS signals. First, prior to data recording, the resistance for each optode probe was monitored and adjusted to meet the minimum criteria defined in the LABNIRS recording software, aiming to achieve a good signal-to-noise ratio [129]. Second, during data recording, the raw optical density (OD) signal in each channel was transformed into concentration changes in HbO and HbR in real time, which were visually inspected to assess signal quality [130]. Third, during pre-processing of the fNIRS signals, we applied an automatic sliding-window detection to further evaluate data quality. Specifically, for each HbO and HbR timeseries, extreme values exceeding mean ± 3 standard deviations (SD) within a 10-second time-window were identified as outliers [131]. Channels with severe motion artifacts or containing more than 5% extreme values were labeled as bad. In order to maintain the same number of channels across all groups, any group with one or more bad channels was excluded from further analyses. Based on these criteria, a total of eight groups were excluded from the final dataset due to poor data quality.”

pages 37-38, lines 1002-1029: “fNIRS signals not only reflected task-evoked brain activity but also systemic physiological interference arising from cardiac pulsation (~1 Hz), breathing rate (~0.3 Hz), and other homeostatic processes [133-136]. Similar to previous studies [6,128], we employed the baseline subtraction approach to mitigate the impact of physiological noise. Specifically, we recorded a resting state session with an identical duration as the task session. As the resting-state predominantly reflects spontaneous hemodynamic oscillations [128], it served as the baseline for comparison. We performed wavelet transform coherence (WTC) analysis on the neural data collected during both the resting-state session (4 min, serving as a baseline) and within-group interaction session (4 min). To reveal the effects of bonding and hierarchy on INS specific to group interaction, we focused on the increased INS during group interaction relative to the baseline (i.e., the resting-state). First, we compared coherence values (averaged across all channels and for each channel) between the within-group interaction session and resting-state session by performing paired-sample t-tests for each frequency (frequency range 0.01-1Hz) [56] to identify the frequency band of interest (FOI, Fig 3A). This analysis identified increased coherence values in two frequency bands: between 0.136 Hz and 0.192 Hz (corresponding to the period between 5.20-7.35s) and between 0.407 to 0.432 Hz (corresponding to the period between 2.31-2.46s). These two frequency bands were chosen as the frequency of interest for the subsequent analyses (FOI, Bonferroni family-wise error (FWE) corrected for multiple comparisons). It is worth noting that no significant results were found in the frequency band of 0.407 - 0.432 Hz (full statistics were reported in S9 Table); therefore, only results based on 0.136 - 0.192 Hz were reported in the main text. This chosen period also effectively captures the temporal structure of the within-group interaction task since one-round within-group messaging typically took an average time of 5-7 s. In addition, this frequency band also excluded high- and low-frequency physiological noises, such as those related to respiration (about 0.2-0.3 Hz), cardiac pulsation (0.7-4 Hz), and high-frequency head movements (> 1 Hz) [128].”

\# Point 11. Were the independent raters ignorant of condition/ study hypotheses?

***Response:*** This is a valid point. We thank Reviewer #1 for asking us to clarify this issue. This should indeed be clear to the reader. Accordingly, in the revised manuscript (page 31, lines 814-818), we have now explicitly stated that the independent raters were blind to experimental hypotheses and conditions.

\# Point 12. Statistical analyses: I was wondering why the authors chose to mainly work with ANOVAs. Would the nested data structure not be better served with LMM?

***Response:*** We greatly appreciated this constructive suggestion provided by Reviewer #1. Per this suggestion, we have applied linear mixed model (LMM) analyses to all statistical testing presented in the main text, and we have fully replicated the main findings. The revised *Results* now included statistics obtained from these LMM analyses.

Our experiment design followed a typical two-factor mixed-model design, with social hierarchy (inter- *vs.* intra-status dyads) as a within-subject factor and bonding (bonding *vs.* control) as a between-subject factor. Consistent with previous studies employing similar data structures (De Dreu et al., 2016; Zhang et al., 2019; Yang et al., 2020), we utilized ANOVA as the conventional and direct statistical analysis approach. In addition, we agreed with the reviewer that LMM is another optimal method for analyzing such structural data. Therefore, in preparing the revision, we performed a series of LMM analyses on behavioral and neural indices, with hierarchy and bonding as fixed effects while considering each group as a random effect (revised *Methods*, page 43, lines 1186-1189), yielding similar results to ANOVA. In the revision, to ensure transparency in reporting our findings, we have chosen to include both sets of results and corresponding statistics derived from these analysis strategies in the revised *Results*.

**Minor:**

\# Point 1. I understand that the authors conducted the study in one cultural context, and it is a great starting point to have collected such an impressive dataset. Yet, I was wondering whether cultural context and culture-specific values might play a role for hierarchical group interactions, especially differential patterns in intra- and inter-status interactions. Perhaps this could be mentioned as an outstanding question for future research in the discussion?

***Response:*** This is an excellent point, and we appreciate the reviewer for raising this question. In response to this point, we have made several clarifications in the revision (page 27, lines 688-710). First, we explicitly clarified that our dataset was collected from participants belonging to one specific culture (page 27, lines 688-689: “it should be noted that the current dataset was obtained from participants of a specific cultural background, i.e., East Asian Chinese individuals.”). Second, we conducted a set of analyses to examine whether cultural values influenced the observed effects. We reported in the revision (page 27, lines 701-706) that our findings were insensitive to culture-specific values. Third, we acknowledged that the absence of modulation by cultural values could potentially be attributed to minimal variability in culture values within a single cultural context. We highlighted the examination of cultural specificity/generality in hierarchical interaction as an outstanding question for future research in the revised *Discussion* (page 27, lines 706-710). Specifically,

**i)** Analysis on the modulation of cultural value on the observed effects

It has been shown that cultural group differences in the neural activity underlying social cognition may be mediated by cultural values, such as interdependence of self-construal (Ma et al., 2014; Hedden et al, 2008). In the current study, we employed the Self-Construal Scale (SCS, Singelis, 1994) to assess individual variation in cultural value of interdependence. Specifically, participants were asked to complete the 24-item SCS before the fNIRS recording to assess their individual differences in interdependent self-construal (higher scores indicating stronger interdependent self-construals). First, we conducted an ANOVA to examine potential differences in cultural values of independence between group leaders and followers. The results indicated no significant differences in cultural values between group leader and followers under in-group bonding and/or no-bonding control conditions (main effect of Hierarchy: *F*_1, 172_ = 1.897, *p* = 0.170, Hierarchy × Bonding interaction: *F*_1, 172_ = 0.147, *p* = 0.702, S1 Table). Second, we performed correlation analyses to examine whether individual variations in cultural values influenced inter- or intra-status interactions in hierarchical groups. This analysis revealed that individual differences in independence did not modulate behavioral and neural indices related to inter- or intra-status interactions nor on the observed behavioral/neural effects (Table R3).

**ii)** Discussion on the culture-related issue

page 27, lines 688-710: “Finally, it should be noted that the current dataset was obtained from participants of a specific cultural background, i.e., East Asian Chinese individuals. This raises the question of whether the observed effects in the current sample can be generalized to individuals from other cultures. Individuals from East Asian cultures place emphasis on group cohesion, interpersonal connection, and social hierarchy [111-113]. In comparison to Western cultures, followers in East Asian cultures tend to display higher levels of obedience and commitment towards their group leader while also encouraging more supportive leadership [114]. Therefore, one may expect cultural differences in how leaders and followers interact, especially after in-group social bonding, in hierarchical groups. To explore this possibility further, we conducted a preliminary examination by assessing individual differences in cultural values within our sample. Previous studies have suggested that cultural group differences in the neural activity underlying social cognition may be mediated by cultural values, such as interdependence of self-construal [115,116]. In the current study, we employed the Self-Construal Scale [117] to assess individual variations in cultural value of interdependence. We found that individual differences in independence did not influence behavioral and neural indices related to inter- or intra-status interactions nor did they affect observed bonding effects on behavioral and neural indices. These results suggested that our findings may be insensitive to culture-specific values. However, we acknowledge that the lack of modulation by cultural values could potentially be attributed to minimal variability in culture values within a single cultural context. It is important for future cross-cultural research to directly test whether and how our current findings can be generalized to other cultural populations.”

- **Responses to Reviewer #2’s comments:**

The current manuscript explores the effects of social bonding on small hierarchical groups, using a combination of behavioral, neuroimaging, and computational methods. The authors recruited 176 participants to form 58 groups of three individuals each, and manipulated social bonding between group members using a color preference task. They then had participants engage in a series of tasks designed to measure group interaction quality, including a group decision-making task and a group competition task.
The current study uniquely combines a set of behavioral manipulations, fNIRS neuroimaging, and applied computational methods with various validations of the findings. The most surprising here is the large sample size, resulting in robust effects. In addition, I must note the clear and well-organized presentation of results, which makes it easy for readers to follow the authors' arguments.
In my opinion, this is one of the best hyperscanning studies that I ever seen!
It should contribute to our understanding of the real-time neurocognitive mechanisms underlying social bonding and may result in further implications for improving team-building and group dynamics in various social contexts.

*Overall response to Reviewer #2’s comment:* We sincerely appreciate the enthusiastic evaluations and constructive suggestions provided by Reviewer #2. In response, we have made revisions in the point-by-point response and to the revised manuscript:

**i)** In the revised *Introduction*, we have reviewed previous hyper-scanning studies related to both symmetric and asymmetric social communications, and provided justification for our chosen regions of interest. **ii)** Additional analyses were conducted to validate the current social-bonding manipulation and address culture-related concerns. **iii)** The revised *Discussion* has been expanded to include a comprehensive discussion of potential limitations. We also discussed cultural specificity/generality and temporal dynamics related to our findings. **iv)** Methodological details for the mediation analysis have been included in the revised *Methods*. We also included both literature justification and behavioral validation of the bonding manipulation in this section. In addition, the figure panels have been modified to display 2 × 2 interactions using only two colors.

A feedback for improvements:

Point #1. Lines 110-111: the authors discuss very briefly previous hyperscanining studies, and the transition to your study looks too sharp. Previous studies have tried to investigate symmetric and non-symmetric social communications. Also, some of the paradigms are close to the current study. For example, you can discuss teacher-teacher vs. teacher-student interactions (e.g. https://doi.org/10.1016/j.bandc.2021.105803), manager-employer interactions (e.g. https://doi.org/10.7358/neur-2019-026-bal2) or maybe you will find even better fit. This way, the transition to the current study should be smoother.
***Response:*** This is an excellent point, and we greatly appreciate this constructive suggestion from Reviewer #2. In the revised *Introduction* (pages 4-5, lines 107-124), we have now reviewed previous hyper-scanning studies related to both symmetric social communications (e.g., among peers or romantic partners) and asymmetric social communications (e.g., between teacher and students, or manager and employee). By incorporating this line of research, the revised *Introduction* flows more smoothly, establishing a stronger connection between prior research and the current study.

pages 4-5, lines 107-124: “Recent neuroscience research has suggested inter-brain neural synchronization as a reliable indicator of social interactions [43-46]. Neural synchronization emerges in a variety of social encounters, including interactions between peers [47], romantic partners [48], parent and child [49-51], teacher and student [52]. The degree of neural synchronization was predictive of interaction quality [53-55]. Of particular interest, neural synchronization among group members has been suggested as a candidate mechanism mediating within-group interaction [21,56,57], and in-group bonding increased within-group neural synchronization [21,58]. However, previous studies merely focused on the egalitarian group, suffering from not being able to differentiate inter- and intra-status interactions and leaving the social bonding effect on hierarchical interactions an open question. Here, we aimed to reveal whether and how social bonding influenced neural synchronization within a hierarchical group and, in particular, the inter-status and intra-status neural synchronization. Recent research has documented stronger neural synchronization during social interactions between individuals with different social roles than those with the same roles (e.g., teacher-student vs. student-student, [59,60]; leader-follower vs. follower-follower, [61,62]). Therefore, it could be expected that in-group social bonding would have differential effects on inter-status and intra-status neural synchronizations.”

Point #2. At the same location (lines 110-111), the authors focus the whole discussion just on the prefrontal cortex. However, IBS in other regions was reported (e.g. rTPJ) and the authors admitted it later. Thus, this part is confusing and not synced with the latter.

***Response:*** We appreciate the reminder from Reviewer #2 and have made appropriate revisions accordingly. Specifically, in the revised *Introduction* (pages 4-5, lines 107-124), the mention of specific regions has been removed from this paragraph. Instead, we discussed the regions of interest (i.e., rDLPFC and rTPJ) in parallel in the following paragraph.

Point #3. You are using only 4-minute length bonding tasks. It souls like to short for social bonding. Please clarify your choice.

***Response:*** We thank Reviewer #2 for asking us to clarify this issue. This is a valid point we did not fully address in the original submission. To clarify this choice, we have included both the literature justification and the behavioral validation of bonding effects as two lines of supporting evidence in the revised manuscript (pages 29-30, lines 759-781; pages 35-36, lines 951-959).

**(i)** First, we have now included the review of key relevant literature to justify the choice of bonding task. The bonding manipulation in the current study was adapted from several previously well-validated procedures (Gaertner & Schopler, 1998; Efferson et al., 2008; Ren et al., 2012; Ma et al., 2015; Yang et al., 2020). Specifically, we merged three essential procedures used in previous studies to manipulate in-group bonding: shared preference (Billig & Tajfel, 1973; Cikara et al., 2011), symbolic marker (McElreath et al., 2013; Efferson et al., 2008), and similarity among group members (Campbell, 1958; Ren et al., 2012). First, for each in-group bonding session, we invited three participants preferring the same color (black or white, as indicated in the online survey) and used this color preference to create group identities, with the individuals referring black over white being labeled as ‘Group Black’ and those preferring white over lack being labeled as ‘Group White’. Second, each participant was then given either a black vest (for ‘Group Black’) or a white vest (for ‘Group White’) to wear during the entire experimental session. The same-colored uniforms serve as an arbitrary symbolic marker and have been proven as a strong approach to enhance group identification, group coordination, and ingroup favoritism (Efferson et al., 2008; Ma et al., 2015). Third, each group engaged in a 4-min online chat session to introduce themselves and identify common features among group members. Brief conversations have been recognized as an effective way to establish and maintain social bond (Kerr & Kaufman-Gilliland, 1994; Clark et al., 2019) and strengthen interconnections among group members, particularly when the topic involved similarities or interdependence among them (Gaertner & Schopler, 1998). However, in the no-bonding control sessions, similar to previous studies (Yang et al., 2020; Ensari et al., 2001), we did not reveal their color preference nor provide a group uniform, and during the 4-min online chatting, individuals were asked to talk about the main courses they had been taking during this semester without being explicitly asked to find shared features.

**(ii)** Moreover, we have analyzed the conversation data and post-experiment rating data (from both participants and independent raters) to examine the effectiveness of the bonding manipulation in the current study. We provided behavioral evidence that ingroup social bonding in the current study efficiently facilitated group communication and fostered positive attitudes towards ingroup members. Specifically, **i)** we showed that social bonding significantly increased group communication by promoting more frequent and responsive interactions within the group (Fig 1D, E; S2 Fig). **ii)** Third-party observers perceived within-group interactions under social bonding as being more frequent and cohesive (S3 Fig). **iii)** Furthermore, we found that social bonding effectively influenced behaviors towards in-group and out-group members: participants under in-group bonding manipulation (compared to no-bonding control) exhibited stronger intergroup discrimination by donating more money to ingroup than outgroup members (Fig 2A), as well as displaying greater self-sacrifice to derogate outgroup members (especially group leader, Fig 2B). **iv)** Finally, participants were asked to rate their impressions and likability of both in-group and out-group members. We found that after undergoing in-group bonding (*vs.* no-bonding control) manipulation, participants expressed higher levels of liking towards ingroup members and reported better impressions of ingroup members (likability: *t*_1, 174_ = 2.943, *p* = 0.009, Fig. R4A; impression: *t*_1, 174_ = 2.929, *p* = 0.004, Fig. R4B). These findings provided statistical support for the effectiveness of our bonding manipulation.

**Fig R4. Bonding effect on subjective evaluations towards ingroup members.** Social bonding increased the self-reported likings towards ingroup members (**A**) and enhanced better impressions from ingroup members (**B**). ***p* < 0.01.

Point #4. Line 875: Please specify what kind of family-wise error rate is used.

***Response:*** We thank Reviewer #2 for this reminder. Accordingly, we have clarified that Bonferroni family-wise error rate was applied in revised *Methods* section (pages 38, line 1021).

Point #5. The authors provide a very detailed description of the statistical methodology for all analyses except the mediation. We should always remember that statistical testing of the mediation is just based on the pattern of correlations in the data. However, the mediation model assumes a causal relationship. Did you try to create any quasi-casual effect using lagging between the mediator and the outcome?

***Response:*** We really appreciated the questions about the methodology and analysis details of the mediation analysis, and the constructive suggestion for additional analyses. Following the reviewer’s suggestion, we have revised the Main Text to provide a detailed description of the mediation analysis (revised *Methods*, page 34, lines 897-911).

Regarding the quasi-casual effect between the mediator and the outcome in mediation analysis, we fully agree with Reviewer #2 that statistical testing of mediation is solely based on correlation patterns in the data and does not provide direct evidence for a causal relationship. We appreciate Reviewer #2’s suggestion of a plausible solution, namely the cross-lagged analysis (Gollob & Reichardt, 1987, Hamaker & Kuiper, 2015), to statistically infer the casual effect between the mediator and the outcome. We examined this approach and assessed its suitability for our current dataset. Unfortunately, as emphasized by Cole & Maxwell (2003), Maxwell & Cole (2007), Hamaker et al. (2015), and Wu et al. (2018), cross-lagged analysis is strongly recommended for testing and establishing mediation with longitudinal data where variables are repeatedly measured over time to allow causal effects to unfold. In our mediation analyses, the independent variable (*X*) was bonding manipulation (bonding vs. control; a between-subjects factor), mediation variable (*M*) was inter-status cohesion, and the dependent variables (*Y*) were social influence/attraction of leader. Both *M* and *Y* were measured only once. Therefore, given our dataset’s characteristics, the cross-lagged analysis method is not suitable for testing causal effects in this particular dataset.

In response to these concerns raised by Reviewer #2, we have taken extra attention in revising *Methods* to avoid any misinterpretation or unwarranted causal inference from our mediation analysis (page 34, lines 911-913).

page 34, lines 897-911: “We next conducted mediation analysis to examine whether the effect of social bonding (the independent variable, *X*) on the leader’s social influence or attraction (the dependent variable, *Y*) was mediated by social cohesion (the mediator, *M*). The mediation analyses were performed using PROCESS (model 4) in SPSS. For each mediation analysis, we computed both the indirect effect (*c*) and direct effect (*c*’), where *c* measures the extent to which *X* influences *Y* through *M*, while *c*’ represents the effect of *X* on *Y* that cannot be explained by *c*. If *c* is nonzero and *c*’ is zero, it indicates full mediation; otherwise, it suggests partial mediation. The significance of mediation was assessed using both Sobel test and Preacher-Hayes bootstrapping. The Sobel test was employed to determine if introducing *M* significantly reduced the relationship between *X* and *Y*. A two-tailed z-test was used to establish statistical significance level. On the other hand, bootstrapping method involved a non-parametric examination of whether zero fell within the bootstrapped 95% confidence intervals (5000 times). If zero did not fall within the confidence intervals, we could conclude that there existed a significant indirect effect.”

page 34, lines 911-913: “It showed be noted that causal direction in the mediation analysis was not directly examined. Further research employing experimental and longitudinal approaches is warranted to validate our current findings from the mediation analysis.”

Point #6. Third-party observers' validation of the interactions is very impressive. I don't think that will make any impact on the results, but inter-rater agreement should be estimated with intra-class correlation (ICC).

***Response:*** Following the reviewer’s suggestion, we conducted the ICC analysis and calculated the absolute agreement (two-way mixed model, average measures) for each rated item. The results of the inter-rater agreement indicated good reliability for the ratings by independent raters (McGraw & Wong, 1996; Koo & Li, 2016). In the revised *Methods* (page 35, lines 943-947), we reported the statistics of ICC analyses on the ratings, including both agreement and consistency.

Point #7. Status-group interactions (2X2). Using 4 colors is pretty complex for reading the graphs. Maybe using just 2 colors (in addition to the x-axis) should be good enough.

***Response:*** We thank Reviewer #2 for this suggestion. Accordingly, we have revised our panels to display 2 × 2 interactions using only two colors.

Point #8. While the authors did a good job of discussing the potential implications of their findings for team-building and group dynamics, it would be helpful to have a more detailed discussion of the potential limitations.

***Response:*** We have incorporated Reviewer #2’s suggestions and provided a more comprehensive discussion of potential limitations in the revised *Discussion* (pages 26-27, lines 667-710).

pages 26-27, lines 667-710: “Our findings may be limited to the current experimental settings and could raise a number of exciting research questions for future studies. First, non-verbal communication, such as gestures, facial expressions, and eye contact, plays a crucial role in real-life social interactions. However, since our participants were restricted to online communication through typing, the effects of social bonding on non-verbal hierarchical interaction remain unexplored. Second, the current study was conducted within simple 3-person hierarchical groups with leaders who represented symbolic, perhaps prestige-style leaders, democratically elected by group members. These leaders made more contributions in intergroup economic games and established positive connections with followers, but lacked the authority to sanction fellow members or allocate resources. These settings deviated from those commonly encountered in complex real-life scenarios. Therefore, caution should be exercised when attempting to generalize our findings and future work is encouraged to explore the generality and specificity of social bonding effects across diverse leadership styles. Third, our study examined the three-person group, which is the minimal unit of a hierarchical group with only two levels of social hierarchy. While group dynamics and leadership in such a small-scale society are arguably representative, it lacks the complexity of group structure institutions. It will be interesting for future research to test whether the bonding effect would be weakened or amplified by the despotic power of non-human animal groups or the complexity of large-scale social networks.

Finally, it should be noted that the current dataset was obtained from participants of a specific cultural background, i.e., East Asian Chinese individuals. This raises the question of whether the observed effects in the current sample can be generalized to individuals from other cultures. Individuals from East Asian cultures place emphasis on group cohesion, interpersonal connection, and social hierarchy [111-113]. In comparison to Western cultures, followers in East Asian cultures tend to display higher levels of obedience and commitment towards their group leader while also encouraging more supportive leadership [114]. Therefore, one may expect cultural differences in how leaders and followers interact, especially after in-group social bonding, in hierarchical groups. To explore this possibility further, we conducted a preliminary examination by assessing individual differences in cultural values within our sample. Previous studies have suggested that cultural group differences in the neural activity underlying social cognition may be mediated by cultural values, such as interdependence of self-construal [115,116]. In the current study, we employed the Self-Construal Scale [117] to assess individual variations in cultural value of interdependence. We found that individual differences in independence did not influence behavioral and neural indices related to inter- or intra-status interactions nor did they affect observed bonding effects on behavioral and neural indices. These results suggested that our findings may be insensitive to culture-specific values. However, we acknowledge that the lack of modulation by cultural values could potentially be attributed to minimal variability in culture values within a single cultural context. It is important for future cross-cultural research to directly test whether and how our current findings can be generalized to other cultural populations.”

Point #9. Are the current findings biased by the cultural context?

***Response:*** In light of this issue, we conducted supplementary analyses regarding the cultural aspect and raised the question of whether the observed effects in the current sample (East Asian Chinese) can be generalized to individuals from other cultures in the revised *Discussion* (page 27, lines 688-710, specific discussion points were included in the response to *Point #8*). We also emphasized that exploring cultural specificity/generality in hierarchical interaction remained an outstanding question for future research.

page 27, lines 688-710: “Finally, it should be noted that the current dataset was obtained from participants of a specific cultural background, i.e., East Asian Chinese individuals. This raises the question of whether the observed effects in the current sample can be generalized to individuals from other cultures. Individuals from East Asian cultures place emphasis on group cohesion, interpersonal connection, and social hierarchy [111-113]. In comparison to Western cultures, followers in East Asian cultures tend to display higher levels of obedience and commitment towards their group leader while also encouraging more supportive leadership [114]. Therefore, one may expect cultural differences in how leaders and followers interact, especially after in-group social bonding, in hierarchical groups. To explore this possibility further, we conducted a preliminary examination by assessing individual differences in cultural values within our sample. Previous studies have suggested that cultural group differences in the neural activity underlying social cognition may be mediated by cultural values, such as interdependence of self-construal [115,116]. In the current study, we employed the Self-Construal Scale [117] to assess individual variations in cultural value of interdependence. We found that individual differences in independence did not influence behavioral and neural indices related to inter- or intra-status interactions nor did they affect observed bonding effects on behavioral and neural indices. These results suggested that our findings may be insensitive to culture-specific values. However, we acknowledge that the lack of modulation by cultural values could potentially be attributed to minimal variability in culture values within a single cultural context. It is important for future cross-cultural research to directly test whether and how our current findings can be generalized to other cultural populations.”

Point #10. Most of the correlations between behavioral and neural variables are very low. They are statistically significant due to the large sample size but should be interpreted with caution.

***Response:*** This is a valid point. Therefore, in the *Method* (page 44, lines 1198-1201), we have clarified that the reported behavior-neural correlations, although statistically significant, should be interpreted and applied with caution due to their small to medium effect sizes (Gignac & Szodorai, 2016).

Point #11. For future studies, it could be great to look at the time trajectories of the behavioral and neural data. Such an approach may provide more insights into the mechanistic characteristics of IBS and social bonding, exploring non-linear effects and running mediation analyses.

***Response:*** We thank the reviewer for raising this insightful question for future studies. First, we attempted to address this question by conducting a preliminary investigation into the time effect on the behavioral and neural data (pages 41-42, lines 1122-1139). We conducted a set of new analyses to explore whether the inter-status interaction pattern and the bonding effect on inter-status interactions were influenced by interaction stages and different time bins (early vs. late). The results showed no significant influence of the interaction-stage or time-bin on behavioral measurements (i.e., turn transitions and response time; Fig R5A, B; Fig R6A, B; Table R4) and neural indices (i.e., INS in the rTPJ, Fig R5C; Fig R6C and INS in rDLPFC, Fig R5D; Fig R6D; Table R4). However, it is important to note that this examination was merely an initial exploration conducted at a coarse time scale, thus unable to unveil non-linear effects or specific neural dynamics at second or millisecond resolution. Therefore, we acknowledge that this needs to be directly and systematically examined in future studies (page 42, lines 1147-1151).

pages 41-42, lines 1122-1139: “To test whether the effects of social in-group bonding on hierarchical interaction were modulated by different interaction stages, we conducted additional analyses. First, we identified the specific time point at which the group leader explicitly emerged in each group. This analysis revealed that the establishment of a group leader occurred approximately halfway through the within-group interaction (with a mean value of 145.60 s, SE = 5.76). Second, based on this identified time point for leader emergence, we divided the within-group interaction session into two stages: pre- and post-leader emergence stages. Third, we conducted 3-way ANOVAs to examine whether these interaction stages influenced our main findings regarding hierarchy and/or bonding effects on both behavioral and neural indices. The results showed no significant impact of the interaction-stage on our main findings (S10A Table). Furthermore, despite observing an average occurrence of leader emergence around the middle of the within-group interaction period, we further balanced the duration between pre- and post-leader emergence stages by dividing it equally into two parts for subsequent analyses. This mid-split approach replicated our observation of no significant impact of different interaction stages (S10B Table). These results suggested that the effects of social bonding on hierarchical interaction remained consistent across different stages of within-group interaction.”

page 42, lines 1147-1151: “We encourage future studies to directly investigate these possibilities. In addition, it is important to note that this examination of time effect was merely an initial exploration conducted at a coarse time scale. Thus, the neural dynamics at second or millisecond resolution needs to be directly and systematically examined in future studies.”

**Fig R5. Null effect of interaction-stage on behavioral and neural results.** The explicit leader emergence time was first identified for each group, based on which, we divided the interaction session into two different stages, i.e., pre-leader emergence and post-leader emergence. Stage × bonding × hierarchy 3way ANOVAs were conducted for each behavioral and neural index. Results showed significant bonding × hierarchy interaction effect on turn transition (**A**) and INS in rDLPFC (**D**), bonding main effect on turn response time (**B**), as well as hierarchy main effect on INS in rTPJ (**C**). While these observed effects were not modulated by interaction-stage (Full statistical reports in Table R4). ***p* < 0.01, ****p* < 0.001. (*Abbr*. inter refers to inter-status; intra refers to intra-status)

**Fig R6. Null effect of time-bin on behavioral and neural results.** To further balance the duration between pre- and post-leader emergence stages, we mid-split the interaction session equally into two parts, i.e., the early and late stage, for subsequent analyses. The results were fully replicated, that is, the bonding × hierarchy interaction effect on turn transition (**A**) and INS in rDLPFC (**D**), bonding main effect on turn response time (**B**), as well as hierarchy main effect on INS in rTPJ (**C**) were not modulated by time-bin. ***p* < 0.01.

Minor: It looks like the color legend for 3B appears in 3C.

***Response:*** Thanks for this reminder. We have revised the figure accordingly.

- **Responses to Reviewer #3’s comments:**

Reviewer #3: Overall, this is an interesting paper that uses rigorous and inventive methods to reveal an intriguing set of results. Hopefully, addressing the questions and concerns below can further strengthen this paper and resolve some potential issues.

*Overall response to Reviewer #3’s comment:* We greatly appreciate the positive evaluations and constructive suggestions provided by Reviewer #3 on our manuscript. Accordingly, we have made significant revisions to address these comments. **i)** In the revised *Introduction*, we have elaborated on key manipulations and provided justification for our chosen regions of interest. **ii)** We conducted additional analysis to validate the current manipulations of social bonding and leadership. Additionally, we performed a set of new analyses to investigate whether the inter-status interaction pattern and the bonding effect on inter-status interactions were modulated by different interaction stages (e.g., before and after leader establishment). We also made significant revisions to the interpretation of the time-lagged results. **iii)** The revised *Discussion* has been expanded to discuss the specificity of our findings in terms of cultural background, leadership context, and group size. **iv)** Two new sections related to leader election and validation were included in the revised *Methods*.

point #1a. In order to improve readers' ability to both understand and assess the findings and potential interpretations thereof, it would be helpful to include more information on key aspects of the the paradigm earlier on - such as how important concepts, such as "social bonding" and "leader" were operationalized.
For example, given that "social bonding" encompasses many possible experiences and activities, it would be helpful to provide more context regarding what "social bonding" entailed in the current study earlier in the paper (e.g., at the end of the introduction, when discussing the current work). Even though the Methods section is at the end of the paper at this journal, this aspect of the paradigm seems very important to clarify to readers to provide context when reading the results. Similarly, it would be helpful to provide more context on this in the paper's abstract.

***Response*:** We appreciate the constructive suggestion from Reviewer #3 to clarify the bonding manipulation earlier in the manuscript. It is indeed necessary to provide this information earlier for the audience. Accordingly, we have briefly addressed how we manipulated in-group social bonding in the revised *Introduction* (page 5, lines 131-135).

page 5, lines 131-135: “The in-group social bonding manipulation employed in the current study was adapted from several previously validated procedures [21,63-66]. Specifically, we integrated three fundamental procedures to manipulate in-group social bonding: i) shared preference [67-68], ii) symbolic marker [64,69], and iii) similarity among group members [65,70].”

point #1b. On a related note, it would be helpful to include more information on how leaders were selected and what differentiated their role. What topics did participants discuss in the online chat when determining who would be a group leader? Did the participants have to discuss the given topic before selecting a leader or could they decide on a leader, then discuss the topic? Was it typically the case that no group member wanted to be a leader and someone was selected at random or asked to be a leader by group members, or was it typically the case that multiple group members wanted to be the leader?

***Response*:** We thank Reviewer #3 for raising these questions, which give us the opportunity to provide further clarifications on the specifics of the leader election (pages 30-31, lines 783-820). Specifically, we have expanded upon the details of leader election in the section titled “Leader election and within-group interaction session” (including the discussion topics and their order; page 30, lines 785-786). Moreover, we have calculated and reported the proportion of groups involving self-nomination (other-nomination), as well as one or multiple leader candidates (page 30, lines 794-798). Furthermore, we have included a new section “Leader election validation” in the revised *Method* (pages 30-31, lines 800-820), where we presented empirical evidence that leaders were not randomly elected or coerced into their positions. We have also validated our experimental setup for establishing the roles of group leader and followers. In the revised *Discussion* (page 26, lines 672-680), we acknowledged a potential limitation that our findings may not be applicable to all leadership situations, particularly those involving formally appointed leaders or leaders with authority to sanction fellows or allocate resources, or facing the risk of replacement.

page 30, lines 783-798: “*Leader election and within-group interaction session.* Participants engaged in a 4-min within-group online chat session. Similar to previous studies [61,80], participants were asked to: i) democratically elect a leader for their group, and ii) discuss the strategies that their group would employ in potential intergroup contests. Participants had the autonomy to decide whether they preferred initiating discussions on strategies, electing a group leader first, or discussing both two topics in parallel. We made it clear to participants that leaders in the current experiment were elected rather than appointed and held symbolic roles without formal power or responsibility over other group members. During the online chatting, participants were labeled by different shapes and communicated via sending messages to remain anonymous (verbal communication was not allowed). For each three-person group, any group member could self-nominate or nominate their peers as the leader. Among all groups, 85% of groups (*N* = 149) nominated one candidate as the leader, who was then approved, while the remaining 15% of groups (*N* = 27) initially nominated multiple candidates but later identified one of them as the final leader later. Of all leaders, 37.5% (*N* = 66) self-nominated while 62.5% (*N* = 110) were nominated by their peers.”

pages 30-31, lines 800-820: “*Leader election validation*. All 176 three-person groups elected their leaders within the 4-min session and successfully recognized group leader in the post-survey. We conducted conversation analysis and compared the total number and length of the utterance given by leader and followers. We found that the group leader was more talkative than followers during group communications (total number of utterances: *t*_175_ = 5.418, *p* = 1.970 × 10^-7^, Cohen’s *d* = 0.408, 95% *CI*: 1.315, 2.822; total length of utterances: *t*_175_ = 8.316, *p* = 2.468× 10^-14^, Cohen’s *d* = 0.627, 95% *CI*: 26.932, 43.693). Moreover, the group leader was more likely to initiate group interaction (leader: 40.3% *vs.* 3-person-average: 33.3%, *p* = 0.039). In addition, we asked participants to report their willingness to be the group leader (0 = not willing at all, 10 = extremely willing). We found that, both in bonding and control conditions, the group leader showed higher willingness and motivation to be the group leader (main effect of leader: *F*_1, 172_ = 64.615, *p* = 1.40×10^-13^, *η^2^* = 0.273). Moreover, leaders who underwent social in-group bonding exhibited a higher level of willingness to be the group leader (leader × bonding interaction: *F*_1, 172_ = 0.067, *p* = 0.812, *η^2^* = 3.31×10^-4^). Finally, the chat transcriptions of all 176 groups were evaluated by independent raters, who were blind to experimental hypotheses and conditions, along domains of i) identifying the group leader of each group (identification accuracy = 97.66%) and ii) reporting perceived interaction quality and leadership. These results suggested that leaders were not randomly elected or coerced into their positions and validated our experimental setup for establishing the roles of the group leader and followers in three-person groups.”

page 26, lines 672-680: “Second, the current study was conducted within simple 3-person hierarchical groups with leaders who represented symbolic, perhaps prestige-style leaders, democratically elected by group members. These leaders made more contributions in intergroup economic games and established positive connections with followers, but lacked the authority to sanction fellow members or allocate resources. These settings deviated from those commonly encountered in complex real-life scenarios. Therefore, caution should be exercised when attempting to generalize our findings and future work is encouraged to explore the generality and specificity of social bonding effects across diverse leadership styles.”

Point #2. Clarifying some of the questions mentioned above about the group interaction/leadership selection phase seem to be very important for understanding the paradigm, for informing interpretations of the results, and critically, for evaluating the appropriateness of the paper's overall framing. The participants have 4 minutes to discuss a given topic and elect a leader of their group, and neural synchrony was assessed during this "group interaction" phase. It seems very important to distinguish between neural synchrony results that pertain to the process of leadership selection vs. conversations among group members after a leader is selected. Thus, it would be helpful to separately analyze these conversations. If some or most of the group interaction time that was analyzed pertain to time *before* a leader was selected and/or during the leader selection process itself, rather than interactions among followers and already-established leaders, this would very significantly change the interpretation of the results and the way that the paper should be framed overall.

***Response:*** We thank Reviewer #3 for the constructive suggestion, which inspired interesting research questions regarding whether i) the inter-status interaction pattern and ii) the bonding effect on inter-status interactions were modulated by different interaction stages (e.g., before and after leader establishment). Accordingly, we performed additional analyses to address these questions. Specifically, we i) identified the time point of leader emergence to split within-group interactions into two stages (i.e., pre- and post-leader emergence) and ii) compared in-group bonding effects on behavioral and neural indices between these two stages. We found that our main findings were not affected by the stage of group interaction and reported these results in the revised *Methods* (pages 41-42, lines 1122-1151). In addition, we provided our interpretation of the null effect of interaction stage, which needs to be directly examined by future studies.

- **Additional analyses**

First, we identified the specific time point when the group leader explicitly emerged in each group. This analysis revealed that the establishment of a group leader occurred approximately halfway through the within-group interaction (with a mean value of 145.60 s, SE = 5.76, i.e., 2 minutes and 25.6 seconds). Based on this identified time point for leader emergence, we divided the within-group interaction session into two stages: pre- and post-leader emergence stages. Second, we conducted 3-way ANOVAs to examine whether these interaction stages influenced our main findings regarding hierarchy and/or bonding effects on both behavioral and neural indices. The results showed no significant impact of the interaction-stage on our main findings, including behavioral measurements (i.e., turn transitions and response time; Fig R7A, B; Table R4) and neural indices (i.e., INS in the rTPJ, Fig R7C and INS in rDLPFC, Fig R7D; Table R4). Furthermore, despite observing an average occurrence of leader emergence around the middle of the within-group interaction period, we further balanced the duration between pre- and post-leader emergence stages by dividing it equally into two parts for subsequent analyses. This mid-split approach replicated our observation of no significant impact of different interaction stages (Fig R8).

- **Our interpretation and discussion**

These results suggested that the effects of social bonding on hierarchical interaction remained consistent across different stages of within-group interaction. Alternatively, it is possible that in the current experimental setting, the group leader implicitly emerged prior to the explicit emergence time point (e.g., during the bonding section). Supporting this possibility, we observed that the group leader was more likely to initiate group interaction at the beginning of within-group interaction (*p* = 0.039) and was already more talkative in the pre-emergence stage (total number of utterances: *t*_175_ = 4.877, *p* = 2.404 × 10^-6^; total length of utterances: *t*_175_ = 7.451, *p* = 4.076 × 10^-12^). Furthermore, a majority of groups (85%, *N* = 149) early on nominated the individual who later emerged as their leader. We encourage future studies to directly investigate these possibilities.

**Fig R7. Null effect of interaction-stage on behavioral and neural results.** The explicit leader emergence time was first identified for each group, based on which, we divided the interaction session into two different stages, i.e., pre-leader emergence and post-leader emergence. Stage × bonding × hierarchy 3way ANOVAs were conducted for each behavioral and neural index. Results showed significant bonding × hierarchy interaction effect on turn transition (**A**) and INS in rDLPFC (**D**), bonding main effect on turn response time (**B**), as well as hierarchy main effect on INS in rTPJ (**C**). While these observed effects were not modulated by interaction-stage (Full statistical reports in Table R4). ***p* < 0.01, ****p* < 0.001. (*Abbr*. inter refers to inter-status; intra refers to intra-status)

**Fig R8. Null effect of time-bin on behavioral and neural results.** To further balance the duration between pre- and post-leader emergence stages, we mid-split the interaction session equally into two parts, i.e., the early and late stage, for subsequent analyses. The results were fully replicated, that is, the bonding × hierarchy interaction effect on turn transition (**A**) and INS in rDLPFC (**D**), bonding main effect on turn response time (**B**), as well as hierarchy main effect on INS in rTPJ (**C**) were not modulated by time-bin. ***p* < 0.01.

Point #3. The time-lagged analyses are especially interesting. However, it is not necessarily the case that the group leaders' brain activity preceding that of followers reflects something that the leader is doing (e.g., "anticipating and predicting the mental stats of followers"), rather than something that the followers are doing (e.g., effectively following the leader). However, this seems to be the only interpretation offered and this interpretation is offered several times. Couldn't it also be that following social bonding, followers are particularly attentive to group leaders, and thus, better at "following" them? The pattern of results shown here does not seem to necessarily entail predictive processing on the part of the leaders.
Point #4. Relatedly, on p. 20, the authors state, "The functional connectivity between rDLPFC and rTPJ has been shown to play a key role in perspective taking, mental inference, and information integrating [74, 75]. We thus expected stronger rDLPFC-rTPJ functional connectivity in leaders than followers, which might account for the leader-to-follower neural alignment." Why would one not have expected followers to also need to engage in perspective taking, mental inference, and information integration? Was this prediction made a priori and if so, what was it based on?

***Response*:** The points #3 and #4, both relevant to the time-lagged analysis, were collectively addressed here. These are valid points and we appreciate Reviewer #3 for bringing them to our attention. Accordingly, we have made significant revisions to the interpretation of the time-lagged results and the hypothesis regarding the relationship between time-lagged neural alignment and rDLPFC-rTPJ functional connectivity.

First, we need to clarify that our focus was on the comparison between leader-to-follower (leader ***proceeding***) neural alignment and follower-to-leader (follower ***proceeding***) neural alignment. Second, we fully agree with the reviewer that leader-to-follower neural alignment can reflect situations where leaders anticipated followers’ mental states as well as situations where followers tracked leader’s mental states. These have been specified in the revised *Results* (page 16, lines 419-422: “The leader-to-follower neural alignment reflects situations in which the group leader leads and followers follow while the follower-to-leader neural alignment may indicate instances where followers lead and group leader follows.”; page 18, lines 462-467: “Taken together ..., indicating that leaders predicted or anticipated followers’ mental states while followers tracked the leader’s mental states to achieve the leader-to-follower neural alignment under the bonding condition.”). Third, according to the reviewer’s suggestion, we replaced the initial hypothesis of “stronger rDLPFC-rTPJ functional connectivity in leaders than followers” with an exploratory analysis in the revised *Results* (page 20, lines 494-499: “As leader-to-follower neural alignment may indicate situations in which group leaders predict followers’ mental states or perspectives [94], as well as when followers strategically attend to and track the group leader [95], we compared rDLPFC-rTPJ functional connectivity between leaders than followers. Furthermore, we tested whether rDLPFC-rTPJ functional connectivity could account for the leader-to-follower neural alignment.”).

**Minor points:**

Point #1. It would be pertinent to discuss the extent to which the current findings would be expected to generalize across contexts and tasks. Relatedly, to what extent do the processes of selecting leaders and exerting leadership here resemble how leadership works outside of the lab? More generally, a discussion of ecological validity would be important.

***Response:*** Thank you for your input. In response, we have considered the suggestion and acknowledged the potential limitations that may arise when applying our findings in real-world scenarios in the revised *Discussion* (pages 26-27, lines 667-686). Specifically, the current study was conducted within relatively simple 3-person hierarchical groups with symbolic leaders who lacked authority to sanction fellow members or allocate resources. These settings deviated from those commonly encountered in complex real-life scenarios; therefore, caution should be exercised when attempting to generalize our results. We have emphasized these constrains and called for future studies to systematically examine our observations within more intricate, large-scale social networks and across diverse leadership contexts.

pages 26-27, lines 667-686: “Our findings may be limited to the current experimental settings and could raise a number of exciting research questions for future studies. First, non-verbal communication, such as gestures, facial expressions, and eye contact, plays a crucial role in real-life social interactions. However, since our participants were restricted to online communication through typing, the effects of social bonding on non-verbal hierarchical interaction remain unexplored. Second, the current study was conducted within simple 3-person hierarchical groups with leaders who represented symbolic, perhaps prestige-style leaders, democratically elected by group members. These leaders made more contributions in intergroup economic games and established positive connections with followers, but lacked the authority to sanction fellow members or allocate resources. These settings deviated from those commonly encountered in complex real-life scenarios. Therefore, caution should be exercised when attempting to generalize our findings and future work is encouraged to explore the generality and specificity of social bonding effects across diverse leadership styles. Third, our study examined the three-person group, which is the minimal unit of a hierarchical group with only two levels of social hierarchy. While group dynamics and leadership in such a small-scale society are arguably representative, it lacks the complexity of group structure institutions. It will be interesting for future research to test whether the bonding effect would be weakened or amplified by the despotic power of non-human animal groups or the complexity of large-scale social networks.”

Point #2. Proofreading would be helpful throughout the manuscript to fix typos (e.g., "In additional") and enhance clarity.

***Response:*** We apologize for the oversight and thank the reviewer for correctly pointing out the typo. Accordingly, we have carefully reviewed the manuscript.

Point #3. No demographic information about participants seems to be provided. It would be helpful to include this.

***Response:*** This is a valid point. A full report of participants’ demographic and psychological information has now been included in the supplementary table (S1 Table, Table R5). Participants in the bonding/control conditions, regardless of their role as leader or follower, did not differ significantly in age, gender, education level, empathic capacity, cooperative personality traits, preference for social hierarchy, cultural values, or baseline intergroup discrimination (all *ps* > 0.05).

Point #4. On a related note, it would enrich the paper to discuss how the current patterns of results might be expected to differ or generalize across cultures and contexts.

***Response:*** In light of this issue, we conducted supplementary analyses regarding the cultural aspect and raised the question of whether the observed effects in the current sample (East Asian Chinese) can be generalized to individuals from other cultures in the revised *Discussion* (page 27, lines 688-710). We also emphasized that exploring cultural specificity/generality in hierarchical interaction remained an outstanding question for future research.

page 27, lines 688-710: “Finally, it should be noted that the current dataset was obtained from participants of a specific cultural background, i.e., East Asian Chinese individuals. This raises the question of whether the observed effects in the current sample can be generalized to individuals from other cultures. Individuals from East Asian cultures place emphasis on group cohesion, interpersonal connection, and social hierarchy [111-113]. In comparison to Western cultures, followers in East Asian cultures tend to display higher levels of obedience and commitment towards their group leader while also encouraging more supportive leadership [114]. Therefore, one may expect cultural differences in how leaders and followers interact, especially after in-group social bonding, in hierarchical groups. To explore this possibility further, we conducted a preliminary examination by assessing individual differences in cultural values within our sample. Previous studies have suggested that cultural group differences in the neural activity underlying social cognition may be mediated by cultural values, such as interdependence of self-construal [115,116]. In the current study, we employed the Self-Construal Scale [117] to assess individual variations in cultural value of interdependence. We found that individual differences in independence did not influence behavioral and neural indices related to inter- or intra-status interactions nor did they affect observed bonding effects on behavioral and neural indices. These results suggested that our findings may be insensitive to culture-specific values. However, we acknowledge that the lack of modulation by cultural values could potentially be attributed to minimal variability in culture values within a single cultural context. It is important for future cross-cultural research to directly test whether and how our current findings can be generalized to other cultural populations.”

Point #5. It appears that the researchers gathered people in-person to do a totally online study. It was a bit confusing when initially reading the paper to discern what parts were in-person and what parts were online, so this would be helpful to clarify early on in the paper.

***Response:*** Per this suggestion, we have clarified in the revised *Introduction* (page 5, lines 128-129) and elaborated in the revised *Methods* (page 29, lines 747-752 that participants were invited to the lab in-person to complete task sessions while simultaneously recording fNIRS neural signals (Fig. 1A snapshots of real experimental sessions). We also explained that we employed online interaction i) to model the increasing virtual interactions that are taking place online nowadays (e.g., online meeting) and ii) to ensure anonymity of each group member, avoiding potential confounding.

Point #6. The rationale for and significance of conversation characteristics (e.g., turn-response time, turn-transition) might not be clear to all readers. Therefore, it could be helpful to discuss this a bit more.

***Response:*** Following the suggestion of Reviewer #3, we have now included the definition, rationale, and significance of conversation measurements in the revised *Results* section (page 6, lines 156-162), aiming to enhance readers’ comprehension of the relevant behavioral measurements.

page 6, lines 156-162: “Turn transition refers to the exchange of utterances among different group members. The number of turn transitions is suggested to reflect the frequency of mutual understanding and engagement [82,83], with more turn transitions indicating more interactive and engaging communications between group members. Turn response time is measured by the time interval between turns, with faster response times reflecting stronger social connection and more efficient and interactive communication [84].”

Point #7. It would be helpful to discuss the rationale for focusing on the right side of the brain.

***Response:*** We appreciate the suggestion from Reviewer #3 and have accordingly specified in the revised *Introduction* (pages 5-6, lines 139-149) the hemisphere information of previous findings. Furthermore, we have clarified that our selection of the right hemisphere’s DLPFC and TPJ as regions of interest was motivated by previous research indicating their involvement in social decision-making and group interaction. Specifically, it has been shown that the right (but not) DLPFC was engaged in allocating attention and making strategic decision during social interaction (Zink et al., 2008; Jeurissen et al., 2014; Rilling & Sanfey, 2011). The TPJ, particularly in the right hemisphere, has been associated with understanding others’ mental states (Young et al., 2010), as well as alignment with in-group members regarding consensus decision and group norms (Bitsch et al., 2018; Saxe & Wexler, 2005). Our choice was also based on earlier studies that have linked neural synchronization in the right DLPFC and right TPJ to social interactive processes (Cui et al., 2012; Lu et al., 2019; Xie et al., 2020) and ingroup cooperation (Yang et al., 2020, Zhou et al., 2022), while highlighting the role of neural synchronization in the right TPJ in leader-follower interaction (Liang et al., 2022; He et al., 2023).

**Table R1. Full statistical reports of the inter-brain neural synchronization and intra-brain connectivity results on HbR signals**

(**A**) Results of Hierarchy × Bonding mixed-model ANOVAs on HbR-INS.

| Channel | Effect | *F* | *p* | *η^2^* | FDR-corrected *p* |
| --- | --- | --- | --- | --- | --- |
| ***TPJ*** |  |  |  |  |  |
| 1 | Bonding | 0.001 | 0.978 | 4.51×10^-6^ | 0.978 |
|  | Hierarchy | 0.365 | 0.546 | 0.002 | 0.846 |
|  | Bonding × Hierarchy | 2.721 | 0.101 | 0.015 | 0.785 |
|  |  |  |  |  |  |
| 2 | Bonding | 3.792 | 0.053 | 0.021 | 0.372 |
|  | Hierarchy | 0.137 | 0.712 | 0.001 | 0.846 |
|  | Bonding × Hierarchy | 1.041 | 0.309 | 0.006 | 0.785 |
|  |  |  |  |  |  |
| 3 | Bonding | 0.165 | 0.685 | 0.001 | 0.978 |
|  | Hierarchy | 4.736 | 0.031 | 0.026 | 0.432 |
|  | Bonding × Hierarchy | 0.130 | 0.719 | 0.001 | 0.785 |
|  |  |  |  |  |  |
| 4 | Bonding | 0.103 | 0.748 | 0.001 | 0.978 |
|  | Hierarchy | 0.170 | 0.680 | 0.001 | 0.846 |
|  | Bonding × Hierarchy | 0.760 | 0.384 | 0.004 | 0.785 |
|  |  |  |  |  |  |
| 5 | Bonding | 3.886 | 0.050 | 0.022 | 0.372 |
|  | Hierarchy | 0.123 | 0.726 | 0.001 | 0.846 |
|  | Bonding × Hierarchy | 0.123 | 0.726 | 0.001 | 0.785 |
|  |  |  |  |  |  |
| 6 | Bonding | 0.015 | 0.903 | 8.63×10^-5^ | 0.978 |
|  | Hierarchy | 0.475 | 0.492 | 0.003 | 0.846 |
|  | Bonding × Hierarchy | 0.670 | 0.414 | 0.004 | 0.785 |
|  |  |  |  |  |  |
| 7 | Bonding | 0.040 | 0.842 | 2.30×10^-4^ | 0.978 |
|  | Hierarchy | 2.855 | 0.093 | 0.016 | 0.650 |
|  | Bonding × Hierarchy | 0.691 | 0.407 | 0.004 | 0.785 |
| ***DLPFC*** |  |  |  |  |  |
| 8 | Bonding | 0.718 | 0.398 | 0.004 | 0.978 |
|  | Hierarchy | 0.304 | 0.582 | 0.002 | 0.846 |
|  | Bonding × Hierarchy | 0.383 | 0.537 | 0.002 | 0.785 |
|  |  |  |  |  |  |
| 9 | Bonding | 0.169 | 0.682 | 0.001 | 0.978 |
|  | Hierarchy | 0.038 | 0.846 | 2.17×10^-4^ | 0.846 |
|  | Bonding × Hierarchy | 0.420 | 0.518 | 0.002 | 0.785 |
|  |  |  |  |  |  |
| 10 | Bonding | 0.160 | 0.689 | 0.001 | 0.978 |
|  | Hierarchy | 0.314 | 0.576 | 0.002 | 0.846 |
|  | Bonding × Hierarchy | 0.923 | 0.338 | 0.005 | 0.785 |
|  |  |  |  |  |  |
| 11 | Bonding | 0.179 | 0.673 | 0.001 | 0.978 |
|  | Hierarchy | 1.847 | 0.176 | 0.011 | 0.821 |
|  | Bonding × Hierarchy | 0.115 | 0.734 | 0.001 | 0.785 |
|  |  |  |  |  |  |
| 12 | Bonding | 0.009 | 0.924 | 5.26×10^-5^ | 0.978 |
|  | Hierarchy | 0.546 | 0.461 | 0.003 | 0.846 |
|  | Bonding × Hierarchy | 0.074 | 0.785 | 4.27×10^-4^ | 0.785 |
|  |  |  |  |  |  |
| 13 | Bonding | 0.112 | 0.739 | 0.001 | 0.978 |
|  | Hierarchy | 0.074 | 0.785 | 4.28×10^-4^ | 0.846 |
|  | Bonding × Hierarchy | 0.353 | 0.553 | 0.002 | 0.785 |
|  |  |  |  |  |  |
| 14 | Bonding | 1.241 | 0.267 | 0.007 | 0.978 |
|  | Hierarchy | 0.116 | 0.733 | 0.001 | 0.846 |
|  | Bonding × Hierarchy | 0.079 | 0.779 | 4.53×10^-4^ | 0.785 |

(**B**) Results of Hierarchy main effect on HbR-FC.

| Channel pairs  (rDLPFC-rTPJ) | *F* | *p* | *η^2^* | FDR-corrected *p* |
| --- | --- | --- | --- | --- |
| **CH8-CH1**** | **8.567** | **0.004** | **0.047** | **0.005** |
| **CH8-CH2*** | **5.587** | **0.019** | **0.031** | **0.021** |
| **CH8-CH3**** | **9.718** | **0.002** | **0.053** | **0.003** |
| **CH8-CH4*** | **4.450** | **0.036** | **0.025** | **0.037** |
| **CH8-CH5**** | **10.234** | **0.002** | **0.056** | **0.003** |
| **CH8-CH6***** | **13.449** | **3.26×10^-4^** | **0.072** | **0.001** |
| **CH8-CH7***** | **18.929** | **2.31×10^-5^** | **0.098** | **1.03×10^-4^** |
|  |  |  |  |  |
| **CH9-CH1**** | **7.953** | **0.005** | **0.044** | **0.007** |
| **CH9-CH2***** | **13.787** | **2.75×10^-4^** | **0.073** | **0.001** |
| **CH9-CH3***** | **20.285** | **1.22×10^-5^** | **0.104** | **6.93×10^-5^** |
| **CH9-CH4***** | **16.761** | **6.48×10^-5^** | **0.088** | **2.27×10^-4^** |
| **CH9-CH5***** | **14.131** | **2.33×10^-4^** | **0.075** | **0.001** |
| **CH9-CH6***** | **23.947** | **2.25×10^-6^** | **0.121** | **2.75×10^-5^** |
| **CH9-CH7**** | **7.953** | **0.005** | **0.044** | **0.007** |
|  |  |  |  |  |
| **CH10-CH1**** | **11.738** | **0.001** | **0.063** | **0.001** |
| **CH10-CH2*** | **6.676** | **0.011** | **0.037** | **0.013** |
| **CH10-CH3*** | **4.970** | **0.027** | **0.028** | **0.029** |
| CH10-CH4 | 1.328 | 0.251 | 0.008 | 0.251 |
| **CH10-CH5**** | **9.556** | **0.002** | **0.052** | **0.003** |
| **CH10-CH6*** | **5.443** | **0.021** | **0.030** | **0.023** |
| **CH10-CH7***** | **13.978** | **2.51×10^-4^** | **0.074** | **0.001** |
|  |  |  |  |  |
| **CH11-CH1**** | **9.693** | **0.002** | **0.053** | **0.003** |
| **CH11-CH2**** | **9.359** | **0.003** | **0.051** | **0.003** |
| **CH11-CH3***** | **29.023** | **2.30×10^-7^** | **0.143** | **1.13×10^-5^** |
| **CH11-CH4***** | **13.983** | **2.50×10^-4^** | **0.074** | **0.001** |
| **CH11-CH5**** | **12.300** | **0.001** | **0.066** | **0.001** |
| **CH11-CH6***** | **25.266** | **1.23×10^-6^** | **0.127** | **2.01×10^-5^** |
| **CH11-CH7***** | **26.450** | **7.23×10^-7^** | **0.132** | **1.77×10^-5^** |
|  |  |  |  |  |
| **CH12-CH1*** | **4.518** | **0.035** | **0.025** | **0.036** |
| **CH12-CH2**** | **7.519** | **0.007** | **0.041** | **0.008** |
| **CH12-CH3***** | **22.629** | **4.11×10^-6^** | **0.115** | **3.77×10^-5^** |
| **CH12-CH4***** | **13.286** | **3.53×10^-4^** | **0.071** | **0.001** |
| **CH12-CH5**** | **10.759** | **0.001** | **0.058** | **0.002** |
| **CH12-CH6***** | **17.970** | **3.64×10^-5^** | **0.094** | **1.37×10^-4^** |
| **CH12-CH7***** | **18.294** | **3.12×10^-5^** | **0.095** | **1.27×10^-4^** |
|  |  |  |  |  |
| **CH13-CH1**** | **10.067** | **0.002** | **0.055** | **0.003** |
| **CH13-CH2*** | **6.615** | **0.011** | **0.037** | **0.013** |
| **CH13-CH3**** | **11.024** | **0.001** | **0.060** | **0.002** |
| **CH13-CH4*** | **5.846** | **0.017** | **0.033** | **0.019** |
| **CH13-CH5**** | **11.479** | **0.001** | **0.062** | **0.002** |
| **CH13-CH6**** | **11.384** | **0.001** | **0.061** | **0.002** |
| **CH13-CH7***** | **21.132** | **8.22×10^-6^** | **0.108** | **5.75×10^-5^** |
|  |  |  |  |  |
| **CH14-CH1***** | **16.494** | **7.37×10^-5^** | **0.087** | **2.41×10^-4^** |
| **CH14-CH2***** | **14.440** | **2.00×10^-4^** | **0.077** | **0.001** |
| **CH14-CH3***** | **22.375** | **4.62×10^-6^** | **0.114** | **3.77×10^-5^** |
| **CH14-CH4***** | **13.209** | **3.67×10^-4^** | **0.071** | **0.001** |
| **CH14-CH5**** | **9.754** | **0.002** | **0.053** | **0.003** |
| **CH14-CH6***** | **19.488** | **1.77×10^-5^** | **0.101** | **8.69×10^-5^** |
| **CH14-CH7***** | **20.193** | **1.21×10^-5^** | **0.104** | **6.93×10^-5^** |

Note: ***** *p* < 0.05, ****** *p* < 0.01, ******* *p* < 0.001, FDR corrected.

**Table R2. Validation of INS increase in real interacting groups**

| Channel | Condition | Effect | *t* | *p* | *Cohen’s d* | *P*  *corrected* | *P*  *permutation* |
| --- | --- | --- | --- | --- | --- | --- | --- |
| ***rTPJ*** |  |  |  |  |  |  |  |
| 3 | Inter-status | **Bonding**** | **3.394** | **0.001** | **0.360** | **0.014** | **0.022** |
|  |  | **Control***** | **3.943** | **1.64×10^-4^** | **0.423** | **0.009** | **0.047** |
|  | Intra-status | Bonding | 0.364 | 0.717 | 0.039 | 0.599 | 0.981 |
|  |  | Control | -0.748 | 0.456 | -0.080 | 0.819 | 0.779 |
| ***rDLPFC*** |  |  |  |  |  |  |  |
| 9 | Inter-status | **Bonding**** | **3.378** | **0.001** | **0.358** | **0.014** | **0.032** |
|  |  | Control | 0.051 | 0.959 | 0.006 | 0.959 | 0.912 |
|  | Intra-status | Bonding | -0.512 | 0.610 | -0.054 | 0.727 | -0.957 |
|  |  | Control | 2.343 | 0.021 | 0.251 | 0.074 | 0.125 |

Note: ***** *p* < 0.05, ****** *p* < 0.01, ******* *p* < 0.001, FDR corrected for 14 recording channels.

**Table R3. Statistical reports of correlation analyses between individuals’ independence-interdependence tendency with behavioral and neural indices in social interaction.**

| Culture-related value | *Social interaction indices* | *r* | *p* | *p(corrected)* |
| --- | --- | --- | --- | --- |
| ***The self-construal scale*** | |  |  |  |
| independence-interdependence difference in leader | Inter-status INS in channel 3 | -0.143 | 0.060 | 0.430 |
|  | Intra-status INS in channel 3 | 0.008 | 0.913 | 0.913 |
|  | Inter-status INS in channel 9 | 0.029 | 0.703 | 0.913 |
|  | Intra-status INS in channel 9 | 0.100 | 0.188 | 0.430 |
|  | Inter-status turn transition | 0.016 | 0.833 | 0.913 |
|  | Intra-status turn transition | 0.101 | 0.187 | 0.430 |
|  | Inter-status response time | -0.122 | 0.112 | 0.430 |
|  | Intra-status response time | -0.109 | 0.158 | 0.430 |
| independence-interdependence difference in follower | Inter-status INS in channel 3 | 0.059 | 0.437 | 0.777 |
|  | Intra-status INS in channel 3 | -0.060 | 0.430 | 0.777 |
|  | Inter-status INS in channel 9 | -0.010 | 0.900 | 0.913 |
|  | Intra-status INS in channel 9 | -0.043 | 0.570 | 0.912 |
|  | Inter-status turn transition | 0.020 | 0.798 | 0.913 |
|  | Intra-status turn transition | 0.122 | 0.110 | 0.430 |
|  | Inter-status response time | -0.152 | 0.047 | 0.430 |
|  | Intra-status response time | 0.035 | 0.653 | 0.913 |

Note: FDR correction.

**Table R4. Statistical reports of interaction-stage/time-bin modulation effects on main findings.**

| Indices in within-group interaction | Examination | Effect | *F* | *p* | *η^2^* |
| --- | --- | --- | --- | --- | --- |
| ***Behavioral*** |  |  |  |  |  |
| Turn transition | **Observe effect**** | **hierarchy× bonding** | **8.395** | **0.004** | **0.046** |
|  | Stage modulating effect | stage × hierarchy× bonding | 0.057 | 0.812 | 3.27×10^-4^ |
| Turn response time | **Observe effect***** | **bonding** | **17.511** | **5.10×10^-5^** | **0.115** |
|  | Stage modulating effect | stage × bonding | 0.134 | 0.715 | 0.001 |
| ***Neural*** |  |  |  |  |  |
| INS in rTPJ (CH3) | **Observe effect**** | **hierarchy** | **10.585** | **0.001** | **0.057** |
|  | Stage modulating effect | stage × hierarchy | 0.529 | 0.468 | 0.003 |
| INS in rDLPFC (CH9) | **Observe effect**** | **hierarchy× bonding** | **11.312** | **0.001** | **0.061** |
|  | Stage modulating effect | stage × hierarchy× bonding | 3.064 | 0.082 | 0.017 |

(**A**)**.** Analysis on pre- and post-leader emergence stages

(**B**). Analysis on early and late time-bin (equally split by mid-point)

| Indices in within-group interaction | Examination | Effect | *F* | *p* | *η^2^* |
| --- | --- | --- | --- | --- | --- |
| ***Behavioral*** |  |  |  |  |  |
| Turn transition | **Observe effect**** | **hierarchy× bonding** | **11.246** | **0.001** | **0.061** |
|  | Stage modulating effect | stage × hierarchy× bonding | 0.064 | 0.800 | 3.71×10^-4^ |
| Turn response time | **Observe effect**** | **bonding** | **10.852** | **0.001** | **0.074** |
|  | Stage modulating effect | stage × bonding | 2.538 | 0.113 | 0.018 |
| ***Neural*** |  |  |  |  |  |
| INS in rTPJ (CH3) | **Observe effect**** | **hierarchy** | **10.204** | **0.002** | **0.055** |
|  | Stage modulating effect | stage × hierarchy | 0.011 | 0.917 | 6.30×10^-6^ |
| INS in rDLPFC (CH9) | **Observe effect**** | **hierarchy× bonding** | **9.280** | **0.003** | **0.051** |
|  | Stage modulating effect | stage × hierarchy× bonding | 2.168 | 0.143 | 0.012 |

Note: ***** *p* < 0.05, ****** *p* < 0.01, ******* *p* < 0.001.

**Table R5. Demographic and psychological information of participants.**

| Measurement | Control | | Bonding | | Bonding | Hierarchy | Interaction |
| --- | --- | --- | --- | --- | --- | --- | --- |
|  | Leader | Follower | Leader | Follower |  |  |  |
|  | Mean (SE) | Mean (SE) | Mean (SE) | Mean (SE) | *p* | *p* | *p* |
| - **Demographic information** | | | | | | | |
| Number (*N*) | 87 | 87 | 89 | 89 | - | - | - |
| Gender (*N_male_* vs. *N_female_*) | 37 vs. 50 | 37 vs. 50 | 43 vs. 46 | 43 vs. 46 | - | - | - |
| Age (year) | 21.40 (0.27) | 21.95 (0.19) | 22.14 (0.29) | 22.16 (0.21) | 0.07 | 0.21 | 0.24 |
| Education year | 16.52 (0.18) | 16.69 (0.14) | 16.92 (0.22) | 16.96 (0.15) | 0.06 | 0.55 | 0.70 |
| - **Psychological information** | | | | | | | |
| Empathic capacity | 2.38 (0.03) | 2.36 (0.03) | 2.38 (0.03) | 2.35 (0.03) | 0.88 | 0.85 | 0.48 |
| Cooperative personality | 3.81 (0.05) | 3.80 (0.04) | 3.84 (0.05) | 3.87 (0.04) | 0.22 | 0.85 | 0.67 |
| Social value orientation | 26.28 (1.38) | 25.52 (0.97) | 25.59 (1.53) | 26.48 (1.05) | 0.82 | 0.94 | 0.59 |
| Prosocial personality | 3.15 (0.04) | 3.19 (0.03) | 3.15 (0.04) | 3.20 (0.04) | 0.23 | 0.96 | 0.89 |
| Justice sensitivity | 2.76 (0.07) | 2.79 (0.06) | 2.69 (0.07) | 2.73 (0.05) | 0.32 | 0.57 | 0.99 |
| Preference for social hierarchy hiehierarchy | 3.43 (0.08) | 3.31 (0.06) | 3.40 (0.09) | 3.41 (0.06) | 0.62 | 0.48 | 0.46 |
| Intergroup discrimination (IDG) | 34.52 (3.83) | 34.23 (2.86) | 35.81 (4.23) | 37.02 (2.82) | 0.56 | 0.90 | 0.83 |
| Ingroup love (IPD-MD) | 43.36 (3.21) | 41.44 (1.91) | 41.78 (3.44) | 46.28 (2.40) | 0.55 | 0.66 | 0.27 |
| Outgroup hate (IPD-MD) | 20.88 (2.45) | 23.91 (1.62) | 24.43 (3.11) | 24.74 (2.13) | 0.36 | 0.49 | 0.57 |
| Independence (SCS) | 5.03(0.07) | 5.05(0.05) | 5.16(0.07) | 4.98(0.06) | 0.58 | 0.24 | 0.15 |
| Interdependence (SCS) | 5.06(0.07) | 5.15(0.05) | 5.14(0.07) | 5.08(0.05) | 0.96 | 0.81 | 0.24 |

**References**

Biro D, Sumpter DJ, Meade J, Guilford T. From compromise to leadership in pigeon homing. Curr Biol. 2006;16(21):2123-8.

Couzin ID, Krause J, Franks NR, Levin SA. Effective leadership and decision-making in animal groups on the move. Nature. 2005;433(7025):513-6.

Podolny JM, Baron JN. Resources and relationships: Social networks and mobility in the workplace. Am Sociol Rev. 1997:673-93.

Zhang H, Gross J, De Dreu C, Ma Y. Oxytocin promotes coordinated out-group attack during intergroup conflict in humans. Elife. 2019;8:e40698.

Hüffmeier J, Wessolowski K, Van Randenborgh A, Bothin J, Schmid‐Loertzer N, Hertel G. Social support from fellow group members triggers additional effort in groups. Eur J Soc Psychol. 2014;44(4):287-96.

Sapolsky RM. The influence of social hierarchy on primate health. Science. 2005;308(5722):648-52.

Ostner J, Heistermann M, Schülke O. Dominance, aggression and physiological stress in wild male Assamese macaques (Macaca assamensis). Horm Behav. 2008;54(5):613-9.

Zink CF, Tong Y, Chen Q, Bassett DS, Stein JL, Meyer-Lindenberg A. Know your place: neural processing of social hierarchy in humans. Neuron. 2008;58(2):273-83.

Jeurissen D, Sack AT, Roebroeck A, Russ BE, Pascual-Leone A. TMS affects moral judgment, showing the role of DLPFC and TPJ in cognitive and emotional processing. Front Neurosci. 2014;8:18.

Rilling JK, Sanfey AG. The neuroscience of social decision-making. Annu Rev Psychol. 2011;62:23-48.

Young L, Camprodon JA, Hauser M, Pascual-Leone A, Saxe R. Disruption of the right temporoparietal junction with transcranial magnetic stimulation reduces the role of beliefs in moral judgments. Proc Natl Acad Sci. 2010;107(15):6753-6758.

Bitsch F, Berger P, Nagels A, Falkenberg I, Straube B. The role of the right temporo–parietal junction in social decision‐making. Hum Brain Mapp. 2018;39(7):3072-85.

Saxe R, Wexler A. Making sense of another mind: the role of the right temporo-parietal junction. Neuropsychologia. 2005;43(10):1391-9.

Cui X, Bryant DM, Reiss AL. NIRS-based hyperscanning reveals increased interpersonal coherence in superior frontal cortex during cooperation. Neuroimage. 2012;59(3):2430-7.

Lu K, Xue H, Nozawa T, Hao N. Cooperation makes a group be more creative. Cereb Cortex. 2019;29(8):3457-70.

Xie H, Karipidis II, Howell A, Schreier M, Sheau KE, Manchanda MK, et al. Finding the neural correlates of collaboration using a three-person fMRI hyperscanning paradigm. Proc Natl Acad Sci. 2020;117(37):23066-72.

Yang J, Zhang H, Ni J, De Dreu CK, Ma Y. Within-group synchronization in the prefrontal cortex associates with intergroup conflict. Nat Neurosci. 2020;23(6):754-60.

Zhou C, Cheng X, Liu C, Li P. Interpersonal coordination enhances brain-to-brain synchronization and influences responsibility attribution and reward allocation in social cooperation. Neuroimage. 2022;252:119028.

Liang Z, Li S, Zhou S, Chen S, Li Y, Chen Y, et al. Increased or decreased? Interpersonal neural synchronization in group creation. Neuroimage. 2022;260:119448.

He Y, Wang X, Lu K, Hao N. Letting leaders spontaneously emerge yields better creative outcomes and higher leader–follower interbrain synchrony during creative group communication. Cereb Cortex. 2023;33(11):6559-72.

Pan Y, Cheng X, Zhang Z, Li X, Hu Y. Cooperation in lovers: an fNIRS‐based hyperscanning study. Hum Brain Mapp. 2017;38(2):831-841.

Liu W, Branigan HP, Zheng L, Long Y, Bai X, Li K, et al. Shared neural representations of syntax during online dyadic communication. Neuroimage. 2019;198:63-72.

Vanzella P, Balardin JB, Furucho RA, Zimeo Morais GA, Braun Janzen T, Sammler D, Sato JR. fNIRS responses in professional violinists while playing duets: evidence for distinct leader and follower roles at the brain level. Front Psychol. 2019;10:164.

Ding XP, Sai L, Fu G, Liu J, Lee K. Neural correlates of second-order verbal deception: A functional near-infrared spectroscopy (fNIRS) study. Neuroimage. 2014; 87:505-514.

Homae F, Watanabe H, Nakano T, Taga G. Prosodic processing in the developing brain. Neurosci Res. 2007; 59(1):29-39.

Taga G, Asakawa K, Maki A, Konishi Y, Koizumi H. Brain imaging in awake infants by near-infrared optical topography. Proc Natl Acad Sci. 2003;100(19):10722-10727.

Zhang M, Mary Ying YL, Ihlefeld A. Spatial release from informational masking: evidence from functional near infrared spectroscopy. Trends Hear. 2018;22, 2331216518817464.

Hoshi, Y. Functional near-infrared spectroscopy: current status and future prospects. J Biomed Opt. 2007;12(6), 062106-062106.

Cui X, Bray S, Bryant DM, Glover GH, Reiss AL. A quantitative comparison of NIRS and fMRI across multiple cognitive tasks. Neuroimage. 2011;54(4):2808-21

Tachtsidis I, Scholkmann F. False positives and false negatives in functional near-infrared spectroscopy: issues, challenges, and the way forward. Neurophotonics. 2016;3(3):031405-.

Ou W, Nissilä I, Radhakrishnan H, Boas DA, Hämäläinen MS, Franceschini MA. Study of neurovascular coupling in humans via simultaneous magnetoencephalography and diffuse optical imaging acquisition. Neuroimage. 2009;46(3):624-32.

De Dreu CK, Gross J, Méder Z, Giffin M, Prochazkova E, Krikeb J, Columbus S. In-group defense, out-group aggression, and coordination failures in intergroup conflict. Proc Natl Acad Sci. 2016;113(38):10524-9.

Ma Y, Bang D, Wang C, Allen M, Frith C, Roepstorff A, Han S. Sociocultural patterning of neural activity during self-reflection. Soc Cogn Affect Neurosci. 2014;9(1):73-80.

Hedden T, Ketay S, Aron A, Markus HR, Gabrieli JD. Cultural influences on neural substrates of attentional control. Psych Sci. 2008;19(1):12-7.

Singelis TM. The measurement of independent and interdependent self-construals. Pers Soc Psychol Bull. 1994;20(5):580-91.

Gaertner L, Schopler J. Perceived ingroup entitativity and intergroup bias: An interconnection of self and others. Eur J Soc Psychol. 1998;28(6):963-80.

Efferson C, Lalive R, Fehr E. The coevolution of cultural groups and ingroup favoritism. Science. 2008;321(5897):1844-9.

Ren Y, Harper FM, Drenner S, Terveen L, Kiesler S, Riedl J, et al. Building member attachment in online communities: Applying theories of group identity and interpersonal bonds. MIS Q. 2012:841-64.

Ma Y, Liu Y, Rand DG, Heatherton TF, Han S. Opposing oxytocin effects on intergroup cooperative behavior in intuitive and reflective minds. Neuropsychopharmacology. 2015;40(10):2379-87.

Billig M, Tajfel H. Social categorization and similarity in intergroup behaviour. Eur J Soc Psychol. 1973;3(1):27-52.

Cikara M, Botvinick MM, Fiske ST. Us versus them: Social identity shapes neural responses to intergroup competition and harm. Psychol Sci. 2011;22(3):306-13.

McElreath R, Boyd R, Richerson P. Shared norms and the evolution of ethnic markers. Curr Anthropol. 2003;44(1):122-30.

Campbell DT. Common fate, similarity, and other indices of the status of aggregates of persons as social entities. Behav Sci. 1958;3(1):14.

Kerr NL, Kaufman-Gilliland CM. Communication, commitment, and cooperation in social dilemma. J Pers Soc Psychol. 1994;66(3):513.

Clark L, Pantidi N, Cooney O, Doyle P, Garaialde D, Edwards J, Spillane B, Gilmartin E, Murad C, Munteanu C, Wade V. What makes a good conversation? Challenges in designing truly conversational agents. In Proceedings of the 2019 CHI conference on human factors in computing systems 2019 (pp. 1-12).

Ensari N, Miller N. Decategorization and the reduction of bias in the crossed categorization paradigm. Eur J Soc Psychol. 2001;31(2):193-216.

Gollob HF, Reichardt CS. Taking account of time lags in causal models. Child Dev. 1987:80-92.

Hamaker EL, Kuiper RM, Grasman RP. A critique of the cross-lagged panel model. Psychol Methods. 2015;20(1):102.

Cole DA, Maxwell SE. Testing mediational models with longitudinal data: questions and tips in the use of structural equation modeling. J Abnorm Psychol. 2003;112(4):558.

Maxwell SE, Cole DA. Bias in cross-sectional analyses of longitudinal mediation. Psychol Methods. 2007;12(1):23.

Wu W, Carroll IA, Chen PY. A single-level random-effects cross-lagged panel model for longitudinal mediation analysis. Behav Res Methods. 2018;50:2111-24.

McGraw KO, Wong SP. Forming inferences about some intraclass correlation coefficients. Psychol Methods. 1996;1(1):30.

Koo TK, Li MY. A guideline of selecting and reporting intraclass correlation coefficients for reliability research. J Chiropr Med. 2016;15(2):155-63.

Gignac GE, Szodorai ET. Effect size guidelines for individual differences researchers. Pers Individ Dif. 2016;102:74-8.
